# On the Sparse Structure of Natural Sounds and Natural Images: Similarities, Differences, and Implications for Neural Coding

**DOI:** 10.3389/fncom.2019.00039

**Published:** 2019-06-26

**Authors:** Eric McVoy Dodds, Michael Robert DeWeese

**Affiliations:** ^1^Redwood Center for Theoretical Neuroscience, University of California, Berkeley, Berkeley, CA, United States; ^2^Department of Physics, University of California, Berkeley, Berkeley, CA, United States; ^3^Helen Wills Neuroscience Institute, University of California, Berkeley, Berkeley, CA, United States

**Keywords:** natural scene statistics, vision, audition, cortex, sparse coding, sensory systems, unsupervised learning

## Abstract

Sparse coding models of natural images and sounds have been able to predict several response properties of neurons in the visual and auditory systems. While the success of these models suggests that the structure they capture is universal across domains to some degree, it is not yet clear which aspects of this structure are universal and which vary across sensory modalities. To address this, we fit complete and highly overcomplete sparse coding models to natural images and spectrograms of speech and report on differences in the statistics learned by these models. We find several types of sparse features in natural images, which all appear in similar, approximately Laplace distributions, whereas the many types of sparse features in speech exhibit a broad range of sparse distributions, many of which are highly asymmetric. Moreover, individual sparse coding units tend to exhibit higher lifetime sparseness for overcomplete models trained on images compared to those trained on speech. Conversely, population sparseness tends to be greater for these networks trained on speech compared with sparse coding models of natural images. To illustrate the relevance of these findings to neural coding, we studied how they impact a biologically plausible sparse coding network's representations in each sensory modality. In particular, a sparse coding network with synaptically local plasticity rules learns different sparse features from speech data than are found by more conventional sparse coding algorithms, but the learned features are qualitatively the same for these models when trained on natural images.

## 1. Introduction

An important goal of systems neuroscience is to discover and understand the principles that might govern sensory processing in the brain. Several principles have been proposed, such as reducing redundancy between neurons (Attneave, 1954; Barlow, 1961; Daugman, 1989; Atick and Redlich, 1992; Chechik et al., 2006), representing statistical dependencies between objects and events to guide action (Barlow, 2001), minimizing expended energy (Laughlin, 2001), maximizing entropy (Schneidman et al., 2006), and maximizing transmitted information (Laughlin, 1981; Bell and Sejnowski, 1995; DeWeese, 1996; Rieke et al., 1997; Hyvärinen and Hoyer, 2001; Karklin and Simoncelli, 2011). Each of these principles suggests that sensory systems should use the statistical structure of sensory data from the animal's environment to efficiently represent and process that data. Studying the statistics of natural sensory input and coding strategies specialized for those statistics has helped us understand neural sensory systems (Dong and Atick, 1995; Bell and Sejnowski, 1997; Schwartz and Simoncelli, 2001; Simoncelli and Olshausen, 2001; Singh and Theunissen, 2003; Olshausen and Lewicki, 2013; Theunissen and Elie, 2014).

One principle that has provided insight into the structure of data from the natural environment and the way these data are represented by neural activity is sparseness (Földiák, 1990; Olshausen and Field, 2004). We say that a fluctuating quantity is sparse if it is often zero (L0 sparseness), or if it is close to zero more often than a Gaussian random variable with the same variance (L1 sparseness). Natural visual scenes can be well-represented by sparse distributions (Field, 1987), and coding strategies optimized for sparseness find local, oriented, bandpass features that match the receptive fields of simple cells in primary visual cortex (V1) (Olshausen and Field, 1996; Bell and Sejnowski, 1997; Rehn and Sommer, 2007; Rozell et al., 2008; Zylberberg et al., 2011). In the auditory domain, the filters that optimize a sparse coding scheme for the acoustic waveforms of natural sounds resemble cat auditory nerve filters, and they form a similar tiling of time-frequency space (Smith and Lewicki, 2006). Interestingly, training this sparse coding model on speech rather than an optimized combination of recordings of environmental sounds yields just as good a fit to auditory nerve filters. Moreover, a sparse coding model of spectrograms of speech learns features that resemble spectro-temporal receptive fields (STRFs) measured at higher stages of auditory processing, such as the inferior colliculus, auditory thalamus, and primary auditory cortex (A1) (Carlson et al., 2012). Some similar features emerge in models of simulated cochlear responses (Klein et al., 2003; Karklin et al., 2012), and hierarchical models have found higher-level sparse structure (Karklin and Lewicki, 2005; Terashima and Okada, 2012; Młynarski and McDermott, 2017). Experiments have uncovered sparse responses from neurons in visual cortex (Vinje and Gallant, 2000; Weliky et al., 2003) and auditory cortex (DeWeese and Zador, 2003; Hromádka et al., 2008) as well as other brain regions (Theunissen, 2003), suggesting that the nervous system has evolved to take advantage of the sparse structure of its inputs. Furthermore, a sparse coding model of natural images exhibits many of the non-classical receptive field effects found in V1 neurons in addition to learning similar classical receptive fields (Zhu and Rozell, 2013).

These results suggest that the applicability of sparse coding to understanding sensory systems is not limited to a single modality, such as vision, but that sparseness may be a more universal property of data from the natural environment. However, there are clear differences between visual and auditory data, which has affected the way they have been explored in past work. For example, sparse coding studies in vision have mostly focused on static images, while the time dimension is not as easily avoided for sounds. As another example, one model designed to separate form and motion in natural movies did manage to learn pairs of phase-shifted Gabor filters (Cadieu and Olshausen, 2012) but it did not learn phase-shifted auditory features, although an extension was used to model binaural sound coding (Młynarski, 2015). Moreover, images exhibit some symmetries (e.g., a rotated natural image is still a natural image) without clear analogs in the auditory domain.

Our primary goal was to compare the statistical structure of natural visual scenes and of natural sounds through the lens of sparse coding. Our approach was to fit complete and highly overcomplete sparse coding models to spectrograms of speech and to natural image patches and then to compare the statistics of these models' representations. We have found that, while natural scenes and sounds can each be well-represented by sparse coding models, this structure differs in significant ways between the two modalities. We focus on the lifetime sparseness of model units, i.e., the sparseness of each unit's activity across stimuli. We also comment on properties related to the sparseness of a model's representation of each stimulus, known as population sparseness.

We further demonstrate that the differences we find between the sparse structure of speech and that of images have significant consequences for coding schemes used to process these types of data, and therefore for neural models of vision and audition. In particular, we study the effects of the statistics of natural sounds and of natural images on a sparse coding network designed to match some important constraints imposed on real neural systems. The Sparse and Independent Local Network (SAILnet) (Zylberberg et al., 2011; King et al., 2013; Zylberberg and DeWeese, 2013) is the only algorithm we are aware of with spiking neurons and synaptically local plasticity rules that can learn the diverse receptive field shapes of V1 simple cells when trained on natural image patches. Some conventional sparse coding algorithms (e.g., Rehn and Sommer, 2007; Rozell et al., 2008) also learn these specific shapes but do not have the same biological constraints. Other algorithms respect some or all of these biological constraints but have not been shown to learn closely matching receptive fields (Savin et al., 2010; Hayakawa et al., 2014; Isomura and Toyoizumi, 2016, 2018) and/or are mechanistically similar to SAILnet (Pehlevan et al., 2015). See section 4.2 for details of the specific “conventional” sparse coding model we used in this paper. We trained SAILnet models on spectrograms of speech sounds and on natural images, using the same preprocessing steps in both cases. While SAILnet learned similar features to those found using conventional sparse coding in the visual case, the SAILnet results were significantly different from conventional sparse coding for auditory data. This divergence in results with SAILnet points to surprising differences between the sparse structure of natural images and natural sounds, with implications for both early development and sensory processing in the mature circuit in these different modalities.

## 2. Results

**Figure 1:**
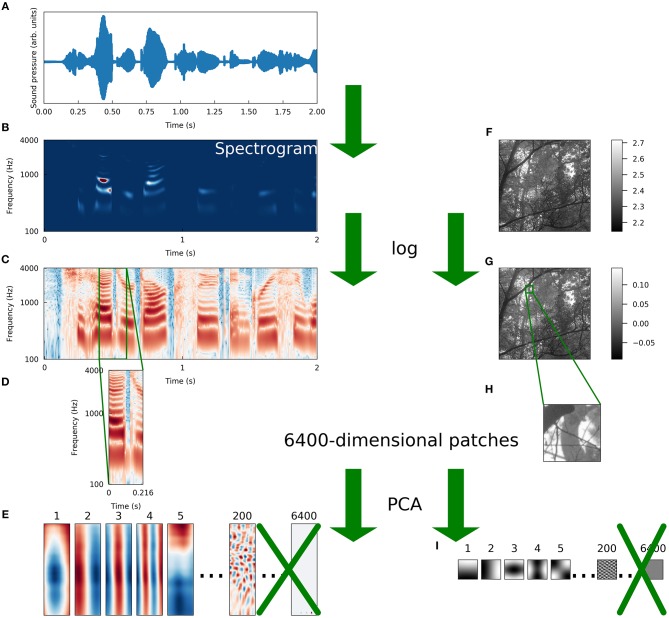
Schematic illustration of preprocessing. We preprocessed a set of natural images and a set of speech sounds using steps as similar as possible to allow for a meaningful comparison of the intrinsic structure of the datasets. **(A)** The raw auditory data consisted of recordings of speech from the TIMIT corpus (Garofolo et al., 1993). The blue curve is the sound pressure waveform of an isolated speaker uttering the first 2 s of “She had your dark suit in greasy wash water all year.” **(B)** Spectrograms were computed from the waveforms (see Methods for details). The color of each pixel represents the intensity (red is more intense, blue is less) of sound at a particular frequency and a particular time. **(C)** We took the logarithm of each intensity spectrogram. **(D)** The spectrograms were divided into overlapping segments of 25 time points each, derived from 216 ms of audio. Since 256 frequencies were sampled at each time point, these spectrogram segments were each 6400-dimensional. **(E)** The dimensionality of the spectrogram segments was reduced by projecting each segment onto the first 200 principal components of that dataset. The variance of each component was then set to one to “sphere” or “whiten” the data. **(F)** Raw image data were taken from the Van Hateren natural image dataset (van Hateren and Schaaf, 1998). The lightness of each pixel represents the intensity of light at that location. **(G)** We took the logarithm of each intensity spectrogram and each intensity image. **(H)** Patches of 80 pixels on each side were taken from the log-intensity images to make 6400-dimensional image patches. **(I)** We repeated the PCA procedure we used for spectrograms exactly on the set of image patches, including whitening.

To compare the sparse structure of speech sounds to that of natural images, we fit sparse coding models to ensembles of each type of data. For speech, we adapted a preprocessing scheme introduced previously (Carlson et al., 2012) in which segments of spectrograms of recordings of speech are first whitened and then reduced in dimensionality using principal components analysis (PCA). We followed the same procedure for image patches of the same dimensionality as the spectrogram segments in order to make as fair a comparison as possible between the two datasets. These preprocessing steps are illustrated in Figure 1 and discussed in more detail in section 4.1. Note that although the preprocessing schemes for the two datasets differed in that we took spectrograms of the auditory data, the spectrogram is not an inherently lossy transformation (Le Roux et al., 2010).

After this preprocessing, we trained sparse coding models using an iterative scheme based on the locally competitive algorithm (LCA) (Rozell et al., 2008) for inference (i,e., determining the activity of each unit for representing a given sensory input) combined with stochastic gradient descent for learning (i.e., setting the parameters of the model). (Note that we will use “activity” and “activation” interchangeably below). Throughout this manuscript we use the term “conventional sparse coding” to refer to this particular scheme, and this is the primary model we used to generate most of the results we present here, but we obtained similar results using SPARSENET (Olshausen and Field, 1996) and, when a comparison made sense, Independent Components Analysis (ICA, Bell and Sejnowski, 1995; Hyvärinen et al., 2001).

### 2.1. Complete Sparse Representations

Before training a sparse coding model, one typically specifies the number of stimulus features (also referred to below as “elements” or “units”) to include in the full “dictionary” of the model. The optimal dictionary learned by a sparse coding model can depend substantially on the size of that dictionary relative to the size of the data (Olshausen, 2013). Intuitively, one might expect a greater diversity of stimulus feature classes with a larger dictionary, and this is often the case. We started by fitting sparse coding dictionaries with 200 elements, which is the dimension of each of our datasets after PCA reduction; we will refer to this as the “complete” regime. While models with more dictionary elements than the dimension of the data may make for a closer correspondence with the brain, we found that the complete regime elucidates some aspects of the datasets themselves that are less clear in the “overcomplete” regime. We also discuss the overcomplete regime in section 2.2. We used the L1-sparse locally competitive algorithm (LCA) (Rozell et al., 2008) to compute sparse codes and stochastic gradient descent (SGD) to optimize the dictionaries (see section 4.2 for details). With a complete dictionary (of which the elements learn to be approximately orthogonal), the differences between LCA and other encoding algorithms have very small effects and distributions of LCA activations primarily reflect the corresponding linear components of the data, whereas non-linearities dominate in the highly overcomplete regime.

**Figure 2:**
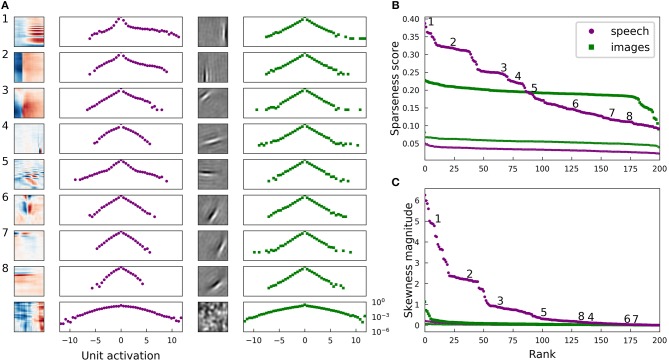
Natural images and speech each exhibit sparse structure, but with clear differences. **(A)** Log-histograms of unit activations for individual elements (units) of complete dictionaries show more skewness and a greater range of sparseness for representations of speech (left pair of columns) than for representations of images (right pair of columns). The sparseness score (Equation 1) can be thought of as a measure of “lifetime sparseness” for the corresponding dictionary element, since it quantifies the sparseness of that element's responses to the range of stimuli it might encounter over its lifetime. Each square contains the spectrogram (left column) or image patch (third column) representing one sparse coding dictionary element fit to the corresponding dataset. For the spectrogram elements, white regions have no effect on the element's activity while red denotes positive weights and blue negative weights. For the image patches, gray represents zero and lighter pixels represent positive weights. Each element has been multiplied by the sign of the skewness of its activations to show the element as it is used by the sparse coding network. The log-histogram next to each element shows how often the unit had a given level of activity. No marker is shown when a histogram bin is empty. The horizontal scale is consistent for each dataset, and the vertical scale is the same for all histograms. The last example for each dataset is not from a trained network but from a network where all elements are independently and identically distributed (iid) Gaussian noise in each principal component after whitening. As expected, these Gaussian noise control cases yield approximately parabolic curves, compared with the more Laplace-like curves for the actual learned dictionary elements. The uppermost examples for speech also exhibit extra weight near zero beyond the peak expected for a Laplace distribution. **(B)** Rank plots for the sparseness score of the distributions of activations of each unit. Each point corresponds to one dictionary element. The numbered points correspond to the numbered examples in **(A)**. Since the activation of one element cannot be determined without the other elements due to the non-linear nature of LCA, these statistics can be meaningfully compared to their values for a full dictionary of random elements (thin curves at bottom). For these curves, we generated 200 random directions in PCA space, used LCA to perform inference on each image patch (or spectrogram segment), and then found the sparseness score of the distribution of activities. **(C)** Rank plots analogous to **(B)** with the magnitude of skewness (Equation 2) substituted for sparseness. The thin curves very close to zero represent the same skewness analysis applied to a collection of 200 random directions in PCA space. The results in all panels are nearly unchanged if unit activations are replaced by linear projections of the dataset onto individual dictionary elements (Figure 7), or for dictionary elements learned with independent components analysis (ICA) (Bell and Sejnowski, 1995) (Figure S5).

Figure 2 illustrates several properties of the learned dictionaries and their representations of the data. The dictionary elements found by our sparse coding algorithm exhibit clear structure beyond the restriction to the subspace spanned by the first 200 principal components. When trained on image patches, the model recovers the Gabor functions and long edge filter-like elements that are known to emerge in sparse coding models of smaller image patches (Olshausen and Field, 1996) (Figure 2A, third column). In the spectrogram case, we recover the element types previously seen in sparse coding dictionaries, including acoustic features that resemble spectro-temporal receptive fields (STRFs) observed in the inferior colliculus and at various other stages of the mammalian ascending auditory pathway (Carlson et al., 2012) (Figure 2A, first column).

For both the visual and auditory case, the distribution of unit activations for every dictionary element was much sparser than is typically found for random directions in the data space. Log histograms of individual unit activities were consistently sharply peaked at 0, and they had fat tails, compared with the parabolic shape of the (log) activity distribution expected for Gaussian-distributed random vectors in the stimulus space (Figure 2A, second and fourth columns).

While both the visual and auditory dictionaries were sparse, there were several striking differences between the sparse structure of their representations. To quantify these observations, we used the following sparseness score:

(1) $$\begin{array}{l} S\left[\left\{y\right\}\right]=-\frac{\langle \left|y\right|\rangle }{\sqrt{\langle y^{2}\rangle }}+\sqrt{2/\pi }, \end{array}$$

where the angle brackets denote the expectation over the empirical distribution of *y*. The constant $\sqrt{2/\pi }\approx 0.80$ simply shifts the score so that a normal distribution has a sparseness score of zero. This measure of sparseness is less sensitive to outliers than is kurtosis (Hyvärinen et al., 2009), for example. Nevertheless, we found qualitatively similar patterns for all of our results using kurtosis (see Figure S6).

Applying this measure to the distribution of activities for a given unit in response to every stimulus in the dataset gives a measure of lifetime sparseness for that unit. Applying this measure to the distribution of activities for a given stimulus over the population of units would instead give a measure of population sparseness.

We found that the lifetime sparseness score was always greater than zero for the learned dictionary elements. For each data set, we then calculated sparseness scores for the activity distributions for a dictionary with each element drawn iid from a normal distribution. These sparseness scores for random elements were small, with median values of 3.3 × 10^−2^ for spectrograms and 5.6 × 10^−2^ for images; these values correspond to a null hypothesis against which to compare the optimized dictionary elements. These control values are plotted as small points in Figure 2B.

While most of the units in the image dictionary clustered around a particular value of sparseness score and appeared qualitatively similar to one another, the units in spectrogram space covered a wider range of sparseness scores, with several distinct clusters (note the plateaus on the left of the purple curves in Figure 2B). These clusters correspond to qualitatively different classes of features: (1) harmonic stacks, (2) broadband onsets, and (3) broadband onsets preceded by high-frequency sound (Figure 2A, first column). Examples of several other qualitatively different types are also shown, although these do not exhibit strong clustering of sparseness scores. The clusters we found resemble those described previously (Carlson et al., 2012) in the usage frequency histogram across the units in a half-complete sparse coding network. These clusters become less distinct as the total number of dictionary elements grows, and they are not apparent from the sparseness scores in the highly overcomplete case considered in the next section. Various other differences in the model or measure used may also make the clustering more or less clear. The complete dictionary is shown in Figure S9, annotated with clusters as determined by fitting a Gaussian mixture model to the sparseness scores and skewness magnitudes.

Another difference between the visual and auditory sparse coding dictionaries was that the auditory unit activations were typically much more asymmetrical compared to the visual units. We quantified this using the absolute value of skewness, which is

(2) $$\begin{array}{l} \left|\text{skewness}\left[\left\{y\right\}\right]|=|\frac{\langle y^{3}\rangle }{{\langle y^{2}\rangle }^{3/2}}\right| \end{array}$$

for mean-centered data {*y*} (Abramowitz and Stegun, 1972). A symmetric data distribution has zero skewness, whereas a distribution with a longer tail on the right than the left has positive skewness. We computed the absolute value of the skewness since, like most sparse coding models, our network allows for both positive and negative activities, leading to degenerate representations of asymmetrical signals. Figure 2C demonstrates that the skewness values for the image dictionary elements were much smaller than the majority of auditory elements. Note also that the three most distinct categories of auditory features cluster in their degree of asymmetry of activations, as measured by the skewness, just as what we found for sparseness.

We can understand the skewness of these elements in terms of properties of speech sounds as represented by power spectra: speech often contains harmonic structure—power concentrated at integer multiples of a fundamental frequency—but it rarely if ever contains the opposite of such structure, which would be broadband sound with power missing at regularly spaced frequencies. Speech, like other natural sounds, also tends to contain sharp onsets but only gradual decays into silence. Since our sparse coding scheme allows for both positive and negative coefficients (i.e., unit activities), we multiplied the examples shown in Figure 2 by the sign of their skewness before displaying them and their corresponding activation histograms, in order to show the acoustic feature that would be added with a positive coefficient to the network's representation of the input. The idea is that the long tail of a skewed distribution of unit activity corresponds to the feature associated with large activity magnitudes; we obtain very similar results if we instead multiply each unit by the sign of its average activity.

The highly sparse and skewed distributions of unit activities onto these well-clustered acoustic feature classes share a distinctive shape exemplified by the first three log-histograms in Figure 2A. In each case, a sharp peak around zero is accompanied by a long flat tail on the positive side, showing that, for example, harmonic stacks appear at a wide range of volumes or not at all. Most of the other activation distributions, for the auditory spectrogram case as for the image cases, have a more symmetric, Laplace-like shape.

We wondered to what extent these results reflected the non-linear inference process of our sparse coding algorithm with 200 interacting elements, as opposed to simply the one-dimensional statistics of the data projected linearly onto each dictionary element. For example, non-linear processing in the retina has been found to be more responsible for decorrelation between retinal ganglion cell outputs than their center surround receptive field shapes, which were originally hypothesized to underlie this effect (Pitkow and Meister, 2012). To address this, we examined the distributions of the training data projected onto individual elements from each of these complete sparse coding dictionaries. Since this is in the complete regime, with no more dictionary elements than there are independent dimensions in the preprocessed dataset, one might expect that the projection of the data onto any given dictionary element (i.e., the distribution of inner products between the dictionary element and the collection of images or spectrograms) should be sparser than projections in random directions, provided our learning algorithm is effective and we have a reasonable model for the data being fit. However, since the dictionary was optimized for the sparseness of codes determined by a non-linear function of the dictionary and the data (LCA, see section 4), it did not have to turn out that linear projections of the data onto every element had to be sparse even if sparse dimensions exist in the data.

Nonetheless, we found that the elements of our optimized complete sparse coding dictionaries did robustly correspond to sparse dimensions in the data (Figure 7). As with the analysis of unit activations, we compared our results for linear projections with those for a dictionary composed of random directions. Specifically, for each data set, we calculated sparseness scores for the distributions of projections for each of 200 directions drawn iid from a normal distribution. As expected, these sparseness scores were small, with median values of 7.8 × 10^−3^ for spectrograms and 2.5 × 10^−2^ for images. For each dataset, the full range of sparseness scores for these 200 random dimensions is represented by the shaded region in Figure 7B, which lies well below the corresponding curve of sparseness scores for nearly all of the dictionary elements learned by the model. As we found for the activity analysis, most of the units in the image dictionary clustered around a particular value of sparseness score and appeared qualitatively similar to one another, whereas the units in spectrogram space covered a wider range of sparseness scores, with several distinct clusters in the high sparseness tail. Moreover, the sparse coefficients determined by our non-linear algorithm were highly correlated with linear projections onto the corresponding dictionary elements with Pearson's *r* = 0.97 for both datasets, and the sparseness statistics evaluated on unit activities correlated with the same statistics evaluated on linear projections with *r*>0.99. The distinction between activations and projections was therefore not important for this analysis applied to these datasets in the complete regime. For a second point of comparison, we also studied dictionaries optimized for the sparseness of linear projections onto the dictionary elements using independent components analysis (ICA). The results are shown in Figure S5 and are also very similar to Figure 2.

### 2.2. Overcomplete Sparse Representations

In addition to our analysis of the complete regime, we also studied the sparse structure of speech and images in the highly overcomplete regime, defined as the case with many more dictionary elements than the dimensionality of the (preprocessed) data. This is particularly interesting from a biological perspective given the greater numbers of neurons in primary sensory cortical areas compared with the number of efferents from the sensory periphery.

In the overcomplete regime, the dictionary elements cannot be truly orthogonal to all other elements, so one might expect non-linear interactions to be more pronounced during inference in order to achieve sparse representations. We fit models with 2,000 elements, which is ten times the dimensionality of the preprocessed data given that we kept only the first 200 PCA components.

**Figure 3:**
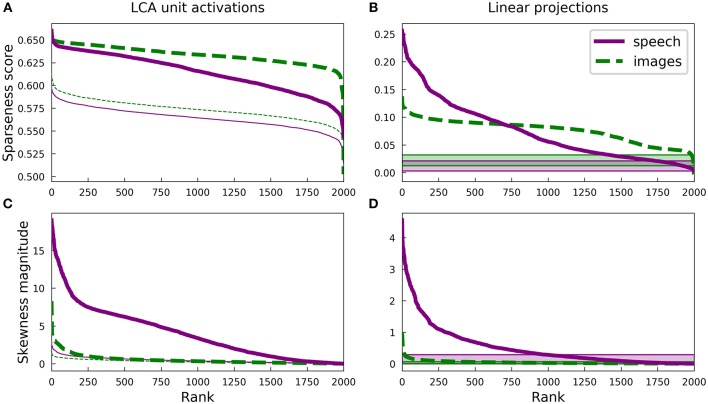
Differences in the sparse structure of natural images and speech are also present in overcomplete representations. As in Figure 2, the various measures presented here are closely related to the lifetime sparseness of individual elements. Ten-times overcomplete sparse coding dictionaries were trained on either the spectrogram dataset or the natural image dataset. Each curve represents 2000 discrete points, with each point representing one element of the corresponding dictionary. **(A)** Sparseness scores were calculated for the activations of the sparse coding dictionary elements using the same L1-sparse LCA algorithm that was used to train the models. To provide meaningful comparisons, the thin curves represent the same analysis applied to 2,000 random directions in the PCA space of the corresponding dataset. As for the complete case shown in Figure 2B, training on speech sounds produced a wider range of sparseness scores across elements. Unlike the complete case, however, the median sparseness was clearly greater for elements from the model trained on images than for the model trained on speech. **(B)** Sparseness scores were calculated for the distribution of each dictionary element's linear projections onto the dataset. Each sparseness score is a statistic of the corresponding dimension of the data space, independent of the sparse coding learning algorithm that was used to find that unit's dimension. As in **(A)** and Figure 2B, the speech dictionary showed a greater range of sparseness values, but unlike **(A)**, there was no systematic difference in the sparseness scores for speech and images. The shaded regions at the bottom show the range of sparseness values one might achieve by chance in the corresponding dataset. Sparseness scores for each dataset were calculated for 2,000 random Gaussian-distributed vectors instead of dictionary elements; the minimum and maximum scores determined the bounds of the shaded region. **(C)** Similar to **(A)** but with skewness magnitudes instead of sparseness scores. **(D)** Similar to **(B)** but with skewness magnitudes instead of sparseness scores.

Figure 3 presents some statistics for the highly overcomplete dictionaries trained on spectrograms and on image patches. Unlike the complete regime, there is no longer obvious clustering of sparseness scores for either unit activations (Figure 2) or linear projections onto the dictionary elements (Figure 7) of the spectrogram dictionary. However, it is still the case that the spectrogram dictionary covers a wider range of sparseness scores than the image dictionary and it has a larger variety of activity distributions (see Figure S2). Intriguingly, the distribution of L0 lifetime sparseness values (i.e., the fraction of stimuli eliciting no response) was nearly identical for the spectrogram and image dictionaries (Figure S4A) unlike what we found for L1 sparseness, though the range of “L0 asymmetry” values (the fraction of positive minus the fraction of negative responses) was still much greater for the auditory model (Figure S4B).

Since LCA uses a non-linear process to determine a sparse representation for each data point and this non-linearity becomes increasingly important for higher degrees of overcompleteness, we examined the sparseness of the activations of each unit in the LCA network and compared it to that of the linear projections for the corresponding unit. The activation of each unit depends on all the units, so we also compared our results to the behavior of a ten-times overcomplete dictionary of random elements (thin lines, Figure 3A; shaded regions, Figure 3B). We adjusted the sparseness parameter λ for each network to achieve the same reconstruction error on the appropriate dataset. For both the image and spectrogram models, the learned dictionary elements had sparser activations than the random dictionary elements of the same rank (Figure 3A), just as we found for the complete regime. Similarly, linear projections were sparser for the learned dictionary elements compared with the random dictionaries for both the image and speech models (Figure 3B). However, the unit activations for the image dictionary were consistently sparser than those of the corresponding spectrogram units (Figure 3A), whereas the sparseness of linear projections (Figure 3B) displayed the same overall pattern we observed for the complete regime, with a larger range of sparseness scores across the spectrogram dictionary compared with a fairly constant middle value for the image dictionary. (Note that the rank (horizontal axis) in each panel of Figure 3 is independently determined).

Thus, unlike what we found for the complete regime, the sparseness of the linear projections of each element of either overcomplete dictionary was not closely correlated to the sparseness of that element's LCA activations: Pearson's *r* of −0.12 and −0.30 for spectrograms and image patches, respectively. Conversely, for both the image and spectrogram models, the skewness of the activations was better explained by the skewness of the linear projections, with Pearson's *r*s of 0.89 and 0.66 (Figures 3C,D). Similar to what we found for the complete regime, the overcomplete spectrogram dictionary exhibited much greater skewness than the overcomplete image dictionary, which was true for both unit activations (Figure 3C) and linear projections (Figure 3D).

These results indicate that the L1 sparseness of the LCA activations in the highly overcomplete regime is strongly affected by interactions among the units and not directly by some aspect of the individual units, while the asymmetry of a unit's activations largely follows from the asymmetry of the corresponding data dimension. This contrasts with the complete regime, where each statistic is nearly the same for linear projections as for LCA activations. Interestingly, these non-linearities increased the sparseness for the overcomplete image model more than for the auditory model (compare Figures 3A,B).

Finally, repeating the analysis described above for L0 sparseness rather than L1 sparseness in the overcomplete regime, we found that most trends were unchanged. For example, both spectrogram- and image-trained networks had much sparser unit activations compared with the random controls (Figure S4A), and the spectrogram activation distributions were more asymmetrical than the image activity distributions (Figure S4B). However, the distributions of L0 sparseness values for images and spectrograms were nearly identical (Figure S4A).

### 2.3. Population Sparseness

The results described above focus on the sparseness of the activations (and linear projections) of a single unit across the dataset, which is directly related to the so-called lifetime sparseness of an individual unit—the distribution of a unit's activities at each moment over its lifetime. We also examined the sparseness of the distribution of simultaneous activations of all units, often called “population sparseness.” These two notions of sparseness are distinct and not always related in an obvious way (Willmore et al., 2011), so it is worth comparing the population sparseness of sound and image models in addition to the lifetime sparseness analyses above.

**Figure 4:**
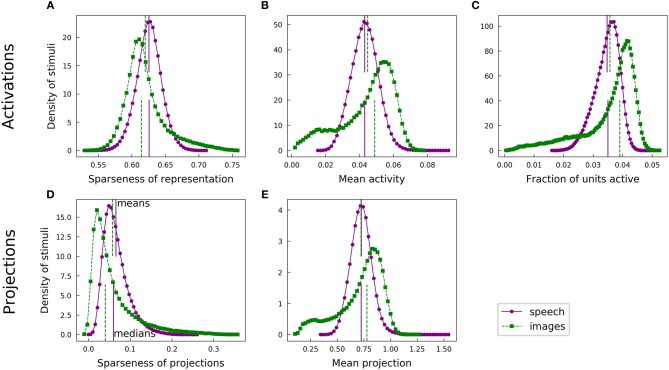
Speech and natural scenes exhibit different distributions of population sparseness for overcomplete dictionaries. **(A)** Sparseness scores (Equation 1) were evaluated on the sparse codes (unit activations) generated by LCA for each spectrogram or image patch in the corresponding dataset. This is one measure of “population sparseness,” which quantifies the sparseness of the full network's representation of individual images or sounds. The purple circles form a histogram of the sparseness scores across the speech dataset, with a linear interpolation plotted in solid purple for clarity. The vertical solid purple lines represent the mean (top) and median (bottom) of the distribution. The green squares constitute the analogous histogram for the image dataset, with a dotted green interpolation curve and vertical dashed lines indicating the mean (top) and median (bottom). Note that, though the means of the two histograms are quite similar, the medians are well-separated, indicating qualitatively that a typical speech sound from our auditory dataset tends to project onto fewer dictionary elements than a typical image patch from our visual dataset. **(B)** Similar to **(A)**, but with the mean activity level replacing the sparseness score. **(C)** The LCA-generated representations had most units completely inactive for any given input (i.e., the L0 sparseness was high). Here we plot histograms of the fraction of LCA units active while representing each image patch or histogram from the relevant dataset. **(D)** Sparseness scores were evaluated on the set of projections of each image patch or spectrogram segment onto the corresponding sparse coding dictionary. These histograms follow the same conventions as in **(A)**. **(E)** Similar to **(D)**, but with the mean absolute value of the projection replacing the sparseness score.

For each of our analyses, a typical speech spectrogram admitted representations with greater population sparseness than did comparably preprocessed images. Each panel of Figure 4 presents a pair of histograms representing comparable distributions over the two datasets. Panels A and B show that the distribution of unit activations representing a given spectrogram segment for an optimized highly overcomplete sparse coding dictionary was typically sparser than the analogous distribution for an image patch. This trend was also evident for the projection analysis (Figures 4D,E). Since LCA uses a thresholding procedure, most units had exactly zero activity for any given stimulus. We therefore also looked at the fraction of units active (a measure of L0 sparseness), which tended to be smaller for the spectrogram case (Figure 4C). Thus, typical elements from the spectrogram dictionary had greater L0 population sparseness, in addition to having greater L1 population sparseness, compared with those from the image dictionary. All of these trends are summarized by the medians of the various histograms, represented by the lower vertical lines in each figure panel.

This observation is somewhat surprising given the opposite trend we found for lifetime sparseness (Figure 3). Speech spectrograms typically admit sparser representations than those of images, even though individual units in the image network tend to have activations with greater sparseness across examples compared to individual auditory units. We emphasize that, while the population sparseness trends we have just described are true for the typical element of each distribution, the distributions for the image case in particular are not fully characterized by a single summary statistic. The means in each plot of Figure 4 are represented by the top vertical lines and the differences are generally small: values of Cohen's *d* were 0.25, 0.16, 0.17, 0.20, and 0.020, for the pairs of distributions in the order of the panels in Figure 4. Normalizing the differences in medians by the same pooled standard deviation as in Cohen's *d* gives magnitudes of 0.52, 0.58, 0.70, 0.49, and 0.35 for the median differences. The distributions for activations and for linear projections show similar differences between the two datasets. This suggests that the effect of the different data statistics on the population sparseness of an optimized sparse coding model is primarily driven by the statistics of the linear projections rather than by complicated non-linear interactions between units during inference.

Although most of our results to this point are robust to preprocessing choices including the use of PCA to whiten and reduce dimensionality, the above comparison of overall levels of sparseness between the datasets depends strongly on these choices. Figures 7, 8 show that alternative image preprocessing leads to substantially different typical levels of sparseness even though the learned dictionary elements and the variation in sparseness are similar.

The population sparseness results for learned dictionaries discussed above all used our trained overcomplete models. Qualitative results are the same for complete models, as shown in Figure S8.

### 2.4. Implications for Biologically Plausible Sparse Coding

Sparse coding dictionaries that resemble the distributions of observed receptive fields of actual simple cells in the primary visual cortex have been obtained using several variations on the classic SPARSENET sparse coding model (e.g., Olshausen and Field, 1996; Bell and Sejnowski, 1997). Among these variations, the Sparse and Independent Local network (SAILnet; a sparse coding model with spiking neurons and synaptically local learning rules) has been shown to learn the variety of simple-cell receptive field shapes seen in primate primary visual cortex when trained on whitened natural image patches (Zylberberg et al., 2011) just as well as the best existing sparse coding algorithms (Rehn and Sommer, 2007; Rozell et al., 2008; Olshausen, 2013). However, we have found that this more biologically plausible sparse coding model does learn a different representation than conventional sparse coding models on some datasets, and that this difference is more pronounced and more clearly relevant to the comparison with real neurons in the auditory case.

**Figure 5:**
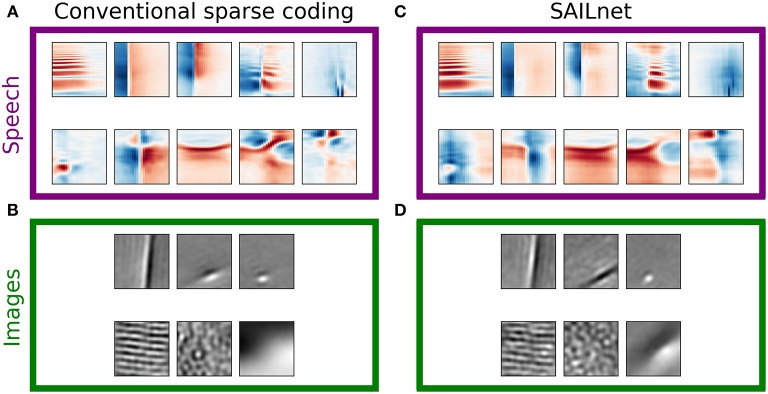
SAILnet and conventional sparse coding learn similar representations when trained on natural images, but not speech. Each box shows elements from a ten-times overcomplete dictionary learned with conventional sparse coding (left) or with SAILnet (right) on one of the datasets. **(A)** For a ten-times overcomplete sparse coding dictionary trained on spectrogram segments, we handpicked elements that show qualitatively different structure. These element types do not occur with equal frequency in the dictionary. **(B)** Elements selected from a dictionary trained on image patches. There are apparently fewer distinct classes of elements in this dictionary than in the speech-trained dictionary. **(C)** SAILnet dictionary elements were selected so as to minimize the angle to each hand-picked sparse coding element. While this yielded similar elements in some cases, there are no elements in the SAILnet dictionary that match several of the dictionary element types seen in the conventional sparse coding dictionary for speech data. **(D)** The SAILnet dictionary trained on images includes good qualitative matches to every element from the corresponding conventional sparse coding dictionary. Full dictionaries are shown in Figures S12, S13, S15, S16.

Figure 5 presents examples of dictionary elements learned by conventional (LCA inference and gradient descent learning) overcomplete sparse coding as described above, each matched with a dictionary element learned by SAILnet on the same data with the same number of dictionary elements. The SAILnet elements were selected automatically to minimize the angle with the corresponding conventional sparse coding element in the 200-dimensional space. The conventional sparse coding dictionary for spectrograms contains elements with no close matches in the SAILnet dictionary, and we were unable to find qualitatively similar elements by inspection in these cases. Full dictionaries are presented in Figures S12, S13, S15, S16. For example, SAILnet does not discover features with the distinct checkerboard structure seen in Figure 5A, second and fifth from the left in the bottom row. These elements tend to have only moderately sparse and mostly symmetric distributions of linear projections on the data (e.g., Figure 2, example 6).

Although we present results for a particular learned dictionary for each dataset and each algorithm, the results do not change substantially for the same algorithm starting from other random initializations and/or using other random draws from the training sets during learning.

**Figure 6:**
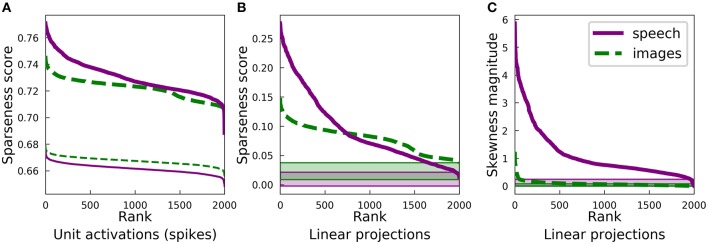
Statistics of overcomplete SAILnet representations are similar to conventional sparse coding for natural images, but they differ for speech. Plots as in Figures 3A,B,D, for ten-times overcomplete dictionaries learned by SAILnet rather than LCA-based learning. **(A)** SAILnet activations are extremely sparse. This plot is analogous to Figure 3A but a direct comparison of the learned dictionaries through these plots is confounded by the differences between LCA and SAILnet inference. **(B)** The sparseness score rank plots qualitatively resemble those for conventional sparse coding (compare with Figure 3B). For the spectrogram-trained dictionary, the lower-rank tail contains somewhat higher sparseness scores than for the conventional sparse coding dictionary. This observation is consistent with SAILnet not utilizing some of the element types conventional sparse coding does (see Figure 5A), since the data's projections onto these element types tend to have relatively low sparseness scores. The shaded regions at the bottom show the range of sparseness values one might observe by chance in the corresponding dataset. Sparseness scores for each dataset were calculated for 2,000 random Gaussian-distributed vectors instead of dictionary elements; the minimum and maximum scores determined the bounds of the shaded region. **(C)** Almost all the SAILnet dictionary elements in the spectrogram case correspond to directions in the data space with large skewness. This is consistent with SAILnet not learning some of the element types shown in Figure 5A, which tend to have symmetric distributions (e.g., Figure 2, example 6). The shaded regions at the bottom indicate the range of possible skewness values one might observe by chance based on the same skewness analysis applied to 2,000 random Gaussian-distributed directions in PCA space for the corresponding dataset.

To understand the differences between the sparse coding dictionaries learned by SAILnet, we examined the sparseness of SAILnet activations after training on each dataset. Figure 6A shows the sparseness of each SAILnet unit, similarly to Figure 3A. Since SAILnet activations are non-negative spike rates, we did not plot the asymmetry of these activations. The thicker lines in Figure 6A represent the activations for trained networks, whereas the thinner lines represent values of sparseness for networks with random dictionary elements (feedforward weights in the SAILnet architecture) after optimizing the other SAILnet parameters at fixed mean spike rate. Interestingly, the trained network had greater sparseness than the network with random dictionary elements, despite the fact that the mean firing rate of each network was fixed to the same value. While some of the qualitative features in Figure 6A agree with those in Figure 3A, others differ. Detailed comparison between these results and those in Figure 3 is hampered by the fact that the two SAILnet networks do not achieve the same reconstruction error, as was the case for the results in Figure 3.

To understand the differences in the learned dictionary elements between conventional sparse coding and SAILnet, we therefore also examined the distributions of linear projections of the data onto the dictionary elements. We found that SAILnet tends to learn stimulus features corresponding to data dimensions that are highly sparse and, when possible, more asymmetrical. Figures 6B,C show rank plots for the sparseness scores and skewness magnitudes of SAILnet dictionary elements projected onto the relevant dataset. These plots are similar in many ways to Figures 3B,D, which show the same statistics for conventional sparse coding dictionaries. The strongest differences are for the spectrogram case: SAILnet learns fewer elements corresponding to data dimensions with low sparseness scores, and almost all of its elements correspond to data dimensions with higher values of skewness than that of any of the 2000 random directions.

The discrepancy between sparse representations for images vs. speech due to skewness can be partly addressed by modifying the SAILnet model to allow for negative spikes. We note that this model with positive and negative spikes is not as biologically-plausible as the original SAILnet model. A complete dictionary learned by this modified SAILnet model is shown in Figure S19. While it learns a few elements with harmonic structure that abruptly reverses sign, a feature found with conventional sparse coding but not the original SAILnet algorithm, this model still does not capture all the features shown in Figure 5A. Furthermore, a dictionary trained with a rectified version of LCA that does not allow negative activities still learns these features. Such a dictionary is shown in Figure S18 and may be compared with the conventional sparse coding dictionary in Figure S12. Thus, the non-negativity of SAILnet can partly, but not entirely, explain the differences between the dictionary elements it learns and those learned in conventional sparse coding models. Full dictionaries for all the models discussed are shown in the Supporting Information.

There are multiple differences between conventional sparse coding models and SAILnet that may appear relevant to the sparse features the models learn. By repeating our basic analyses with other modifications of SAILnet, we determined that the spike-based coding scheme does not noticeably affect the results discussed above but that the local learning rules for the dictionary elements and lateral connections are the crucial difference.

### 2.5. Alternative Image Preprocessing

Although we attempted to make as fair a comparison as possible between the sparse structure of images and spectrogram representations of speech, there is no clear natural or canonical mapping between these datasets to which we could appeal to justify our handling of the data prior to fitting sparse models. We therefore also studied an alternative scheme of “preprocessing” that has been common in the literature since the seminal sparse coding work of Olshausen and Field (1996). These image patches are 16x16 pixels and have been approximately whitened by applying a filter to the original images.

**Figure 7:**
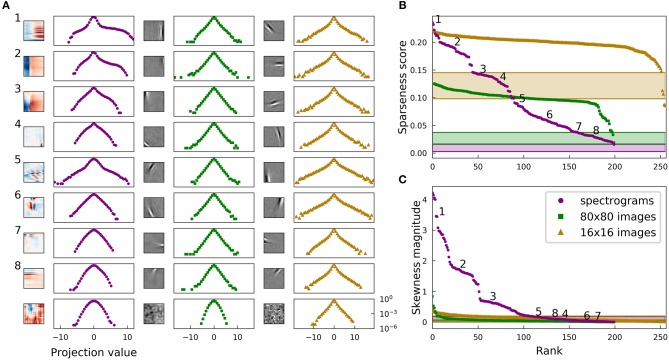
Histograms of projections exhibit the same sparse structure as unit activities for both natural images and speech in the complete regime. Filter-whitened images exhibit greater sparseness. This figure is identical to Figure 2, but with unit activities replaced by linear projections of individual dictionary units on the corresponding dataset, and the addition of a third dataset consisting of filter-whitened 16 × 16 image patches [rightmost pair of columns in **(A)**, beige curves in **(B,C)**]. Note that the results for our primary datasets look very similar to those in Figure 2, demonstrating that projections and activities exhibit very similar statistics in the complete regime, even for different preprocessing of the image dataset. Results for the filter-whitened images are similar to those for the PCA-whitened data except that random directions in the space of filter-whitened images are already quite sparse and so learned directions are still more sparse, as discussed in section 2.5. The shaded regions at the bottom of **(B,C)** show the range of sparseness or skewness values one might observe by chance in the corresponding dataset; sparseness scores (skewness values) on each of the three datasets were calculated for 200 Gaussian-distributed random vectors instead of dictionary elements, and the minimum and maximum scores (values) determined the bounds of the shaded region for the corresponding dataset. All results in this figure are for linear projections and not sparse codes represented by unit activities generated by a non-linear coding algorithm. However, the results are similar for dictionaries learned with independent components analysis (ICA), in which case the activity corresponding to each dictionary element is itself a linear projection of the stimulus (Figure S5).

Figure 7 shows sparseness and skewness plots and example distributions for complete sparse coding on the filter-whitened images, expanding Figure 2 to include results on this third dataset. Figure 7 also differs in that it presents the statistics for linear projections of the dictionary elements onto the elements of the datasets. As discussed in section 2.1 and as can be seen by comparison to Figure 2, this distinction is minor in the complete regime.

Random directions in the space of 16 × 16 images whitened as in Olshausen and Field (1997) are fairly sparse, with median sparseness score 0.13. This fact is well-known and largely accounted for by variation in the local variance of natural images (Baddeley, 1996; Lyu, 2011). The trend of excess sparseness of the sparse coding dictionary closely follows the trend for the other image dataset. While a fair comparison of overall levels of sparseness between datasets clearly becomes difficult when preprocessing is not matched exactly, we believe our primary comparisons of the structure associated with sparseness in the datasets are robust to these preprocessing choices.

For the PCA-reduced datasets, we used 10-times overcomplete sparse coding, while for the 16 × 16 image patches we used a network that was nominally 8-times overcomplete, making it about 10-times overcomplete given that some dimensions are essentially noise. Our results on this dataset closely match those shown in Olshausen (2013). A dictionary is shown in Figure S14, and sparseness and skewness rank plots are shown in Figure S3.

The sparseness of an overcomplete dictionary element's linear projections is not closely correlated to the sparseness of that element's LCA activations for this dataset: Pearson's r of −0.16. The skewness of the activations is better explained by the skewness of the linear projections, with Pearson's *r* = 0.59. These observations qualitatively echo what we saw for the other datasets.

**Figure 8:**
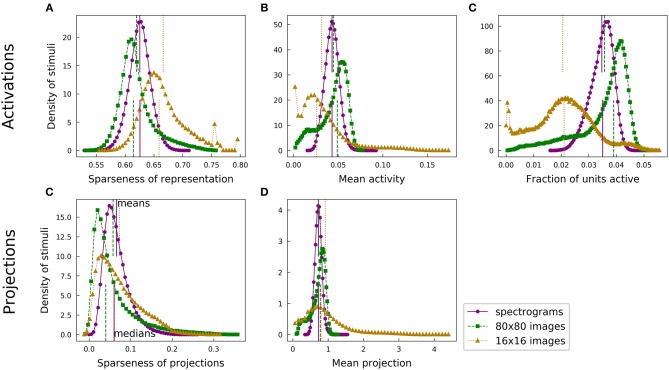
Distributions of population sparseness statistics across each dataset, including filter-whitened images. This figure is identical to Figure 4 except for the addition of a third dataset consisting of filter-whitened 16 × 16 image patches (beige). Direct comparison of the filter-whitened images' statistics with those of the other datsets is made difficult by the differing whitening schemes, as discussed in section 2.5.

The fact that the 16x16 image patches are not fully whitened hampers meaningful comparisons among the various population sparseness results. Plots are shown in Figure 8. The filter-whitened images generally admit sparser representations, but the effect is partly driven by the whitening scheme rather than by the intrinsic structure of the data.

A simple argument demonstrates why whitening should matter, particularly for the population sparseness of optimized sparse codes as we computed them. In an extreme case, the data variance may be so much greater along one dimension than all others that it is possible to achieve 15 dB SNR reconstructions with only the special dimension non-zero. Then only one unit need be active, in which case the population sparseness of the representation is ≈0.8 by our measure (the precise value depends on the dictionary size) for most data examples. The same data, after whitening, does not permit this trick since no direction has more variance than any other. The filter-whitened images are not exactly whitened, and the residual variation in the variance of different dimensions allows a model to obtain greater population sparseness than if the data were fully whitened. Imperfect whitening can also strongly affect the features found by SAILnet—an interesting topic for future work.

Other differences between the preprocessing schemes may also confound comparisons; this is why we attempted to use as similar as possible preprocessing for our primary datasets.

## 3. Discussion

Guided by the principle of sparse coding, we have explored the statistical structure of natural stimuli from two different sensory modalities, vision and audition. Both natural images and natural sounds admit sparse linear representations, but we have found some clear differences.

For complete sparse coding models trained on natural image patches, the lifetime sparseness of individual features was nearly uniform across the learned dictionary, reflecting the uniform sparseness of the linear projections of the dictionary elements onto the image dataset. Complete dictionaries trained on spectrograms of speech, however, showed a much wider range of lifetime sparseness values, both in terms of unit activations and projections, although the average sparseness was comparable for the two models. Moreover, the spectrogram dictionary included many units with highly asymmetric distributions of activity (and projections) across the dataset, unlike the highly symmetric distributions displayed by the image dictionary elements. We also find that these statistics exhibit a degree of clustering in the spectrogram case. There are several qualitatively different spectrotemporal shapes, and dictionary elements with certain shapes tend to have similar values of sparseness and skewness magnitude. These shapes include harmonic stacks, broadband onsets, and lower-frequency onsets preceded by high-frequency sound. There are a number of other qualitatively different elements in the complete spectrogram dictionary at lower levels of sparseness, but these do not cluster as clearly.

Most of these trends persisted in the highly overcomplete regime, but we found that the lifetime sparseness of unit activations was greater for the image dictionary, unlike population sparseness, which was typically greater for the spectrogram dictionary. The clustering of statistics for distinct spectrogram dictionary element types is no longer apparent in the overcomplete regime, possibly due to a greater number of elements blurring the clusters together or to the lifetime sparseness statistics being more determined by non-linear interactions with other units and therefore less closely tied to the individual elements. The spectrogram dictionary elements still covered a wider range of sparseness scores, excepting one outlying image dictionary element. We observed this effect in all similar analyses with the exception of using an L0 sparseness measure in the overcomplete regime as shown in Figure S4, where the two modalities led to a similar small range of L0 sparseness values.

We then compared the distribution of visual features learned by a biologically-plausible sparse coding model called SAILnet trained on images with the distribution of acoustic features obtained when the model was trained on speech spectrograms. Despite the strong agreement between the visual features learned by SAILnet and those learned by more conventional sparse coding models, the spectrogram dictionary produced by this model differed markedly from the set of acoustic features learned by conventional sparse coding models. While SAILnet recovers some qualitatively similar sparse features, there are some classes of sparse features that conventional sparse coding finds but SAILnet does not. For example, conventional sparse coding finds localized “checkerboard” shapes as useful sparse features while SAILnet does not. We found that part of the discrepancy between the features learned by SAILnet and those learned by conventional methods could be resolved by modifying the SAILnet model to allow negative spikes, but that some differences, including the “checkerboard” example just mentioned, remained. The key difference between SAILnet and conventional methods is the use of synaptically local learning rules. It may be that the sparse structure of speech sounds is less amenable to such learning rules, compared to images.

While we endeavored to make the comparison between the sparse structure of images and that of sounds as fair as possible, the raw datasets are quite different and the preprocessed datasets may still differ in some ways that confound our comparisons. We have included results in the Supplementary Material using an alternative image preprocessing scheme to show what may change as the data is handled differently. Our primary interest is in the structure of the data associated with sparseness, as captured by sparse modeling. The learned dictionary elements and their variability in sparseness and skewness is remarkably similar between the two preprocessed image datasets despite large differences in the construction of these datasets (including in image patch size, whitening method, and dimensionality reduction). However, typical values of lifetime and population sparseness are significantly greater for the image dataset with alternative preprocessing as described in section 2.5. It is not clear which differences matter here, although the different whitening schemes play some role. This observation serves as a caveat on our results reported in section 2.3 comparing typical population and lifetime sparseness values across modalities.

Previous studies have made comparisons between the statistics of natural visual and acoustic data and their implications for neural coding in these modalities. Well-known examples of this include the fact that natural scenes and sounds both exhibit power spectra with power law functional forms (Field, 1987; Attias and Schreiner, 1997) and natural scenes obey spatial translational invariance just as natural sounds obey time translational invariance. One property shared by visual and auditory responses is the gain dependence modeled by divisive normalization (Schwartz and Simoncelli, 2001). Recent work has also shown that a model that minimizes neural wiring while efficiently representing stimuli learns various subcortical receptive fields in the visual and auditory systems (Shan et al., 2016).

However, while sparse coding has been remarkably successful at predicting the receptive fields of V1 simple cells based on the structure of natural scenes, there is not yet a comparable result for primary auditory cortex (A1), despite the apparent sparse structure of natural sounds. That a linear sparse coding model can represent natural scenes at all is perhaps surprising given the highly non-linear processes, such as occlusion by opaque objects and cast shadows, that cannot be explicitly represented by linear summation models. Conversely, raw acoustic waveforms are actually very close to linear summations of different individual component sounds in the environment. Consistent with this, previous work has demonstrated success with sparse coding at subcortical stages of the auditory system. A sparse coding model trained on raw auditory waveforms learns to tile time-frequency space in the same way as cat auditory nerve fiber filters measured by reverse correlation (Smith and Lewicki, 2006), but this model applies to the auditory nerve—the earliest stage of auditory processing once acoustic signals are converted into spike trains. Sparse coding models of non-linear spectrogram or cochleogram (Lyon, 1982; Slaney, 1998) representations can learn sparse structure on longer time scales (Klein et al., 2003), and some of the learned dictionary elements resemble the diverse STRF shapes found at various stages of the ascending auditory pathway (Carlson et al., 2012), including the inferior colliculus (ICC), the medial geniculate body (MGB) of auditory thalamus, and even some neurons recorded in A1, but across the dictionary the agreement is not as strong for any brain region as has been demonstrated for V1 (Olshausen and Field, 1996; Rozell et al., 2008; Zylberberg et al., 2011).

This dichotomy in the ability of sparse coding models to fully capture response properties of neurons in V1 vs. A1 could reflect the possibility that A1 and V1 are not directly analogous, even if they are both primary sensory cortices. If we include the visual processing taking place in the retina, there are roughly equal numbers of processing stages in the visual and auditory pathways leading to A1 or V1, as quantified by the number of synaptic connections needed to reach each of these cortical areas (although the auditory system has more subcortical areas along the way). However, due to the greater dimensionality of visual input (the two optic nerves are comprised of roughly 10^6^ axons and there are ≈10^8^ photoreceptors, whereas there are fewer than 10^5^ fibers in the two cochlear nerves) and strong non-linearities, such as occlusion affecting visual input, it may be that more stages of processing are required for visual signals to reach the same level of refinement as auditory representations in A1. This is qualitatively consistent with the greater number of visual cortical areas compared with the number of auditory areas.

The aspects of the sparse structure of natural sounds that differ from the structure of natural images could guide our pursuit of better models of the relevant auditory brain regions. Our analysis points to some relevant considerations. One is that the asymmetry between greater and lesser sound intensity is important, especially for biologically realistic models restricted to have non-negative activations. In addition, the sparseness of individual features optimized to form sparse representations of spectrograms of speech vary widely compared to the relatively uniform sparseness of sparse visual features. Moreover, dependencies among the activities of units in overcomplete dictionaries—which are most relevant for biology—influence which dimensions in stimulus space are most useful for sparse coding. A concrete manifestation of this is that a network, such as SAILnet, in which units cannot cooperate directly based on the knowledge of other units' contributions to the coding, will not learn some of the same acoustic features as a network, such as an LCA-based scheme, in which such cooperation is explicitly incorporated. The inter-unit connections in SAILnet, learned with only information locally available at the synapse, are more biologically plausible, but they lead to different behavior. In the auditory case, the differences include learning a more limited sparse coding dictionary that does not match as many receptive fields measured in real neurons. This observation suggests that SAILnet may need to be modified to better account for auditory sparse coding. More generally, the dependence on the stimulus statistics we observe for a biologically plausible model suggests that some properties of neural coding need to be specialized for the auditory system, even though it may share the basic principle of sparse coding with the visual system. A biologically realistic mechanism for finding approximate solutions to an optimization principle may be effective for one type of data, but not for another.

Indeed, SAILnet was specifically designed to model learning and inference in V1. In particular, it treats different orientations within its two-dimensional input on an equal footing, which makes sense given that these are all spatial dimensions in the visual case. In fact, the algorithm does not assume any special relationship between the various pixels—one could scramble their locations or convert the pixel array into a vector with any ordering and SAILnet would find the same features when mapped back to the unscrambled space. There is typically some mild anisotropy present in natural images (e.g., vertical and horizontal edges are often slightly over-represented compared with random orientations), but this could be learned by using the same learning rules in all orientations in the two-dimensional image space. Spectrograms, however, are strongly and inherently anisotropic, with time represented along one cardinal axis and (a non-linear function of) acoustic frequency along the other. Perhaps this contributed in some way to the divergence between our SAILnet results for speech spectrograms and what we found using conventional sparse coding, but if so this is a subtle effect given that the LCA-based sparse coding algorithm we used also employs isotropic rules for learning and inference. We emphasize that, even though SAILnet may not treat time in a natural way for a biologically-realistic mechanistic model of auditory processing, it provides a useful tool for identifying aspects of the sparse structure of natural sounds that differ from those of natural scenes.

Motivated by previous work (Lewicki, 2002; Smith and Lewicki, 2006), we analyzed speech data as a proxy for a more complete collection of “natural sounds.” As recapitulated here, spectrograms of speech by themselves have a rich sparse structure, with several distinct feature types that our models use for their sparse codes; some of these features resemble STRFs measured in inferior colliculus and other brain regions (Carlson et al., 2012). Using speech is particularly convenient, since using ensembles of recorded sounds has been shown to yield good agreement between sparse coding predictions and auditory nerve response properties, such as the same time-frequency trade-off, only when the relative proportions of three different types of recorded sounds are empirically adjusted to fit the model (Lewicki, 2002; Smith and Lewicki, 2006). Thus, using speech data is in some sense a more principled approach, since it removes two adjustable parameters from the model.

This picture is somewhat complicated by the fact that the filters learned in such models depend on the sound class used even when the time-frequency tiling properties match (Lewicki, 2002). It is also unlikely that speech captures the structure of natural sounds that occurs on the longer time-scales of our spectrogram-based models. To address this, we fit sparse coding models to the ensemble of natural sounds described in Smith and Lewicki (2006). The sparse structure captured by our models in that data is less rich than, and largely redundant with, what we found for speech. We have included a dictionary and sparseness rank plot in Figures S7, S20.

More broadly, what constitutes the relevant ensemble of “natural scenes” or “natural sounds” is not clear to us; these notions may not be well-defined or independently determinable in a way that does not rely on fitting neural response properties. Another question is whether or not one can determine definitively if a given type of natural signal is truly sparse with the sort of analysis employed here. In particular, preprocessing using PCA or some other dimensionality reduction technique necessarily changes the structure of the data for any realistic scenario (i.e., unless the raw signal is strictly L0 sparse, with the relevant dimensions contained within the space spanned by those PCA dimensions retained for later analysis).

There are persuasive arguments challenging the notion that natural scenes or sounds are truly sparse, in the L0 sense, for the sort of linear generative models we have considered here (Lyu and Simoncelli, 2009; Hénaff et al., 2015).

In addition, the oriented filters learned by sparse coding models represent a shallow optimum, as representations of natural scenes using center-surround filters, for example, are almost as sparse (Bethge, 2006; Eichhorn et al., 2009).

There are good reasons to question (Lyu and Simoncelli, 2009) why the local oriented filters predicted by sparse coding models trained on natural images would first appear in V1, skipping past the highly non-linear retina and lateral geniculate nucleus (LGN), if indeed sparseness is the correct normative principle for the visual system. Moreover, the sparseness of successive sensory representations at higher stages of processing in the ascending auditory (Chechik et al., 2006) and visual (Rust and DiCarlo, 2009, 2012) pathways does not always appear to increase.

There exist alternative choices for the models as well as the data, and different models yield different results. The results from SAILnet learning differ enough from those from gradient descent learning to be of interest, but we did not observe any substantial differences between the results of gradient descent learning using different algorithms to compute the sparse codes. Furthermore, while a greater variety of sparse features of natural images than found in early sparse coding work (e.g., Olshausen and Field, 1996) has been shown using various methods, we are not aware of any work showing sparse features of natural images that do not have qualitative matches in a 10-times overcomplete conventional sparse coding network. We believe that our approach at least captures the known sparse structure of natural images in terms of feature diversity and so can be taken as representative of the subtly varying results that different sparse coding and learning algorithms uncover.

In this work, we have taken a pragmatic approach to our model selection and data choices. Undoubtedly, the specific sparse coding models we have employed here are imperfect approximations to whatever model would best fit ensembles of natural scenes and sounds as defined by our datasets, but by applying these models to both images and sounds, we have been able to identify several similarities and differences between the statistical structure of these natural signals. We are, of course, motivated by the fact that the sparse coding models we consider here can predict receptive fields in V1 and several cell types at various stages of the ascending auditory pathway, even if these models do not entirely capture the statistics of natural signals. It will be interesting in future studies to explore more fully the structure of natural stimuli, and its implications for neural coding.

Beyond the particular results presented in this work, we have shown that it is possible and fruitful to compare the sparse structure of natural data from different modalities. The principle of sparse coding appears to have applicability to auditory data as well as visual data, supporting the idea that sparseness is, to at least some degree, a universal property of natural data. Nonetheless, we have found that there are aspects of sparse structure that are clearly not universal. Understanding these differences offers insights into the structure of natural stimuli and into the ways in which neural systems represent it.

## 4. Methods

### 4.1. Data

We performed our primary analyses on three sets of natural data; Figure 1 illustrates the preparation of the two primary datasets we compared. The same preprocessing steps were taken where possible, in order to reveal the effects of the structure inherent in the data rather than differences in how the data were presented to the sparse coding algorithms. In addition to these two comparably prepared datasets, we used an image dataset preprocessed by methods common in the literature to reveal some effects of this processing. Results from this alternative image dataset are discussed in section 2.5.

Following previous work (Klein et al., 2003; Smith and Lewicki, 2006; Carlson et al., 2012), we focused on human speech as a rich class of natural sounds. Speech data were taken from the TIMIT continuous speech corpus (Garofolo et al., 1993) and preprocessed as in Carlson et al. (2012). Specifically, we divided each waveform by 10 times its variance and removed any DC value. We then used MATLAB's (MATLAB, 2016) spectrogram function to calculate the discrete Fourier transform (DFT) of Hamming-windowed segments of 16 ms (256 samples) of sound, with neighboring segments overlapping by half their length. The DFT was sampled at 256 frequencies logarithmically spaced between 100 Hz and 4 kHz. We trimmed the power spectrograms to remove periods of silence and then took the logarithm of the results. We divided these spectrograms into overlapping 25-timepoint (216 ms edge-to-edge) segments, yielding about 3 × 10^5^ spectrogram segments. While this procedure is not a precise model of early auditory processing, previous work has found better agreement with experimental data using spectrograms than with preprocessing meant to emulate the cochlea (Carlson et al., 2012). Spectrograms also provide a representation often used for generating stimuli and visualizing spectro-temporal receptive fields in the experimental literature (e.g., Miller et al., 2002; Qiu, 2003; Fritz et al., 2005; Rodríguez et al., 2010; Theunissen and Elie, 2014). Although using only the (log) power obscures the phase structure, the original sound waveforms can in fact be reconstructed from power spectrograms using implicit phase structure from overlapping windows (Le Roux et al., 2010).

Natural image data was taken from a subset of the van Hateren database (van Hateren and Schaaf, 1998) with minimal blur and other artifacts (see Olshausen, 2013). Using other grayscale natural image datasets, such as that of Olshausen and Field (Olshausen and Field, 1996) has not been seen to produce drastically different results in sparse coding. We extracted ≈3 × 10^5^ 80-by-80 pixel patches from the images and took the logarithm of the intensity at each pixel. The mean log-intensity was removed from each patch.

The speech spectrogram segments and the natural image patches were both 6,400-dimensional, and we used PCA to reduce the dimensionality to 200. We also divided each principal component by its variance, achieving a “whitened” or “sphered” representation in which the empirical covariance matrix was equal to the identity matrix (Kessy et al., 2015). The PCA step discarded about 7% of the variance in each of the two raw datasets. Another 18% of the original variance in the images was removed by the patch-wise mean subtraction described above. No comparable effort was made to remove the dimension of largest variance in the spectrogram data, following (Carlson et al., 2012). After whitening, this dimension had the same variance as the others and therefore did not strongly affect our results.

Discarding an equal amount of variance does not guarantee equality in the degree to which dimensions are important to the sparse structure of the data have been discarded. Our comparisons are, more precisely, between the sparse structure of linear subspaces of the datasets, which will not perfectly reflect the sparse structure of the datasets themselves. The dimensionality reduction in both datasets corresponds roughly to low-pass filtering with a hard cutoff, in spatial-frequency space for images and temporal- and frequency-modulation frequency space for spectrograms. The approximate scale invariance of natural images (Ruderman and Bialek, 1994; Zoran and Weiss, 2009) suggests that the subspace should reflect the sparse structure at a particular scale or range of scales, as long as the range of spatial frequencies retained is large enough. Our results suggest that this is the case, although some minor ringing artifacts from the hard cutoff are visible in the image patches and learned dictionary elements (see full dictionaries in the Supplementary Figures). In the case of spectrograms, the dimensionality reduction discards fine-grained details that may be of secondary importance to an organism processing the sounds. For example, we are able to understand speech reconstructed from our first 200 principal components. Regardless of the importance of the discarded components, though, it is possible that some of our comparisons between the linear subspaces do not generalize to the unreduced data.

Our choices were driven by the need to make the two datasets comparable, so our preprocessing differed from that employed in much of the literature. We repeated our analyses on a third dataset, containing the same natural images as they were preprocessed in Olshausen (2013) and other sparse coding work. There were two key differences: first, Olshausen (2013) used small image patches of 16 × 16 pixels while we used larger patches of 80 × 80 pixels. Since Olshausen (2013) first downsampled by a factor of 2, the scale of our images is better compared to 32 × 32 patches. Since natural images have less variance in higher spatial frequencies, our dimensionality reduction also discarded the information destroyed by this downsampling.

The other crucial difference between these two image datasets is due to the whitening step. Olshausen and Field (Olshausen and Field, 1996, 1997; Olshausen, 2013) whitened their raw images using a filter that flattens the Fourier spectrum at low frequencies while allowing the variance of very high frequencies, which is largely noise, to remain small. In contrast, we exactly equalized the variance of the first 200 principal components and removed the other components entirely. Results with the images preprocessed as in Olshausen (2013) are discussed in section 2.5.

Reconstructions of original data from our reduced representations are shown in Figure S21.

### 4.2. Sparse Coding

Sparse coding is a probabilistic model with latent variables *a*_*m*_ whose prior distribution is factorial with each factor given by the same sparse distribution (in this work, a Laplace distribution):

(3) $$\begin{array}{l} p_{a}\left(a\right)\propto \underset{m}{\prod }e^{-\lambda |a_{m}|}, \end{array}$$

where λ is a parameter that determines the width of the distribution and therefore how strongly the prior favors sparse sets of *a*_*m*_. These latent variables serve as the coefficients in linear combinations of a set of vectors Φ_*m*_ that in this work we call “dictionary elements.” Each such linear combination, plus some Gaussian noise, corresponds to a data example, such as an image: $x_{i}=\underset{m}{\sum }a_{m}\Phi_{mi}+n_{i}$.

The *a*_*m*_ are determined by maximum *a posteriori* (MAP) inference given input data *x*.

The *a*_*m*_ estimated in this way are often referred to as the activity of the *m*th unit, and the dictionary elements Φ_*m*_ are often compared to receptive fields of neurons. The analogies to neurons suggested by these terms are not exact, but a unit's dictionary element is approximately the same as the linear receptive field that would be measured for that unit with an activity-triggered average (Olshausen and Field, 1996).

The dictionary elements Φ_*m*_ are learned by stochastic gradient descent on the model log-likelihood.For each step, the gradient is averaged over a minibatch of 100 data examples.

The use of MAP inference requires that we constrain the norms of the Φ_*m*_ to prevent solutions with small *a*_*m*_ and large, meaningless Φ_*m*_. We therefore divide each Φ_*m*_ by its norm after each gradient step. Using the MAP estimate to compute gradients for learning is not guaranteed to result in the same learned dictionary Φ, but a method that uses more samples from the posterior learns familiar Gabor functions on whitened natural image patches (Theis et al., 2012).

### 4.3. Locally Competitive Algorithm

We used the L1-sparse locally competitive algorithm (LCA) (Rozell et al., 2008) to perform MAP inference. LCA uses a dynamical system with auxiliary variables that are thresholded to obtain estimates of *a*^MAP^. Typically most of the auxiliary variables are below threshold and the $a_{m}^{\text{MAP}}$ estimates are exactly zero for most *m*. The threshold is set by the sparseness parameter λ. We dynamically adjusted this parameter to achieve reconstructions with 15 dB signal-noise ratio while training the models, allowing direct comparison to the results of Olshausen (2013).

The choice of coding algorithm is not crucial to our results, and learning using alternative inference schemes yields similar dictionaries. This is particularly true for dictionaries that are not overcomplete, as demonstrated by the similarity of the results in Figure S5, which used Independent Components Analysis (ICA) (Bell and Sejnowski, 1995), to Figure 2, which used LCA and stochastic gradient descent on the mean-squared error.

### 4.4. SAILnet

We used the Sparse and Independent Local network (SAILnet) model (Zylberberg et al., 2011) to study how the statistics of different stimuli interact with biologically realistic constraints. SAILnet uses spiking neurons and synaptically local plasticity rules to achieve sparse codes. Mathematically, SAILnet can be understood as optimizing the Lagrange function

(4) $$\begin{array}{l} L=\frac{1}{2}\underset{mi}{\sum }{\left(X_{i}-\Phi_{mi}a_{m}\right)}^{2}+\underset{m}{\sum }\theta_{m}\left(a_{m}-p\right)+\frac{1}{2}\underset{mn}{\sum }W_{mn}\left(a_{m}a_{n}-p^{2}\right). \end{array}$$

Here the first term approximates the mean-squared error in the sparse coding log-likelihood in the limit that the *a*_*m*_ are sparse and uncorrelated. Maximizing with respect to the Lagrange multipliers θ_*m*_ and *W*_*mn*_ constrain the *a*_*m*_ to be sparse, with average activity *p* ≪ 1, and uncorrelated. The *a*_*m*_ are the firing rates of leaky integrate-and-fire circuits with thresholds θ_*m*_ and inhibitory connections between neurons with strengths *W*_*mn*_. The dynamics of this circuit approximately seek firing rates *a*_*m*_ that minimize L. As in conventional sparse coding, the dictionary elements Φ_*mi*_ are updated at fixed *a*_*m*_ using; the Lagrange multipliers are updated at the same time but with greater rates to ensure the constraints are satisfied during learning.

The SAILnet Lagrange function, and in particular the approximation to mean-squared error in the first term of Equation (4), allow the gradient descent update for each connection to be computed using only information available at that connection, e.g., one only needs to know *a*_1_ and *a*_2_ to update *W*_12_. The cost of this locality is that SAILnet units do not directly learn to cooperate to represent the data.

Although SAILnet has been shown to learn the expected dictionary Φ on whitened natural images, in some ways it behaves differently from a conventional sparse coding algorithm, such as LCA with gradient-descent based learning. Here we have focused on how SAILnet interacts with differing input statistics.

### 4.5. Model Implementation

We implemented soft-thresholded LCA (Rozell et al., 2008) in TensorFlow (Abadi et al., 2015) to learn the overcomplete sparse coding dictionaries. We implemented SAILnet in Python. Code for these implementations may be found online at github.com/emdodds/DictLearner and github.com/emdodds/SAILnet. For the ICA results shown in Figure S5 we used the FastICA (Hyvärinen and Oja, 2000) implementation in scikit-learn (Pedregosa et al., 2011).

## Data Availability

No datasets were generated for this study. Natural image data from van Hateren and Schaaf (1998) and speech data from Garofolo et al. (1993) were used.

## Author Contributions

ED and MD conceived, designed the study, wrote and revised the manuscript. ED performed the analysis.

### Conflict of Interest Statement

The authors declare that the research was conducted in the absence of any commercial or financial relationships that could be construed as a potential conflict of interest.
