# Supplementary material for: On the Sparse Structure of Natural Sounds and Natural Images: Similarities, Differences, and Implications for Neural Coding
Source: Front Comput Neurosci. 2019 Jun 26;13:39. doi: 10.3389/fncom.2019.00039 (PMC6606779; doi:10.3389/fncom.2019.00039)
Supplement: Supplementary file 1 [file Data_Sheet_1.pdf]

Supplementary Material: On the sparse structure of natural sounds and natural images: similarities, differences, and implications for neural coding

PRIMARY FIGURE LABELS FOR REFERENCE

Figure 1.

Figure 2.

Figure 3.

Figure 4.

Figure 5.

Figure 6.

Figure 7.

Figure 8.

1 SUPPLEMENTARY TABLES AND FIGURES

1.1 Figures

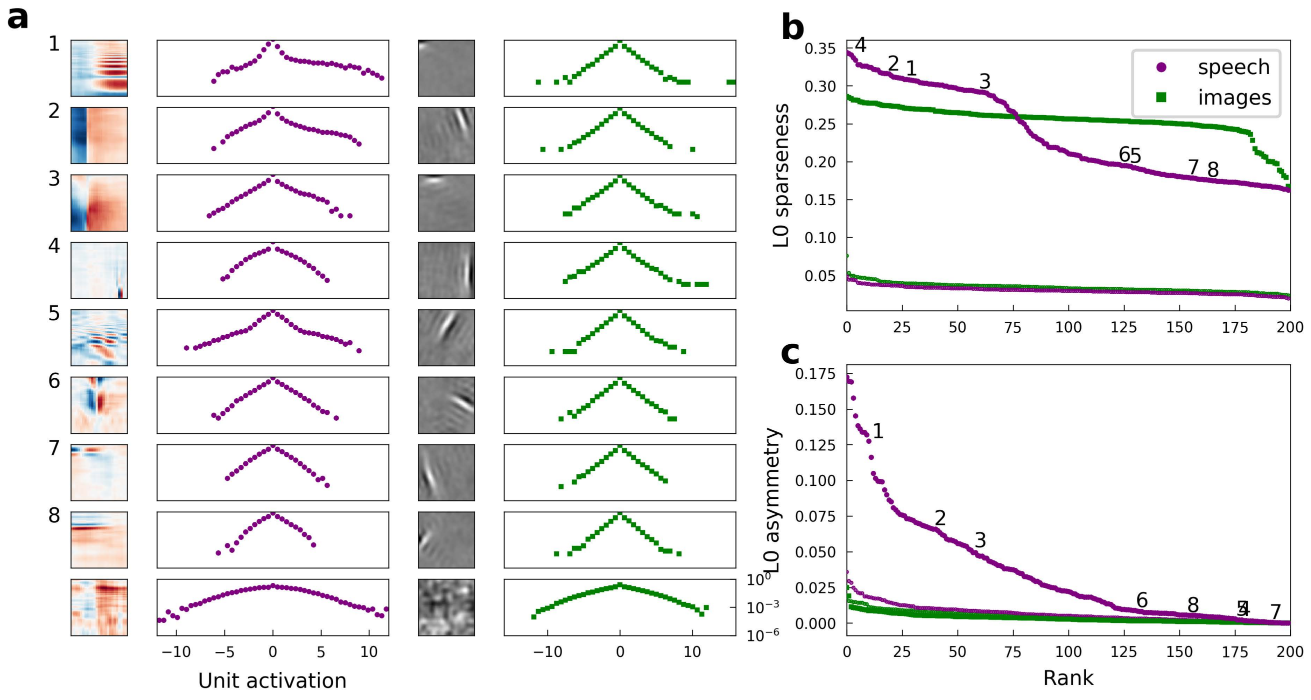

Figure S1. L0 lifetime sparseness rank plots for complete sparse coding This figure is identical to Fig. 2 except that panels b and c show statistics that disregard the magnitude of activations. The L0 sparseness of a unit is the fraction of stimuli that did not elicit a nonzero activation from the unit. The L0 asymmetry is the absolute value of the difference between the fraction of stimuli that elicited a positive response and the fraction that elicited a negative response.

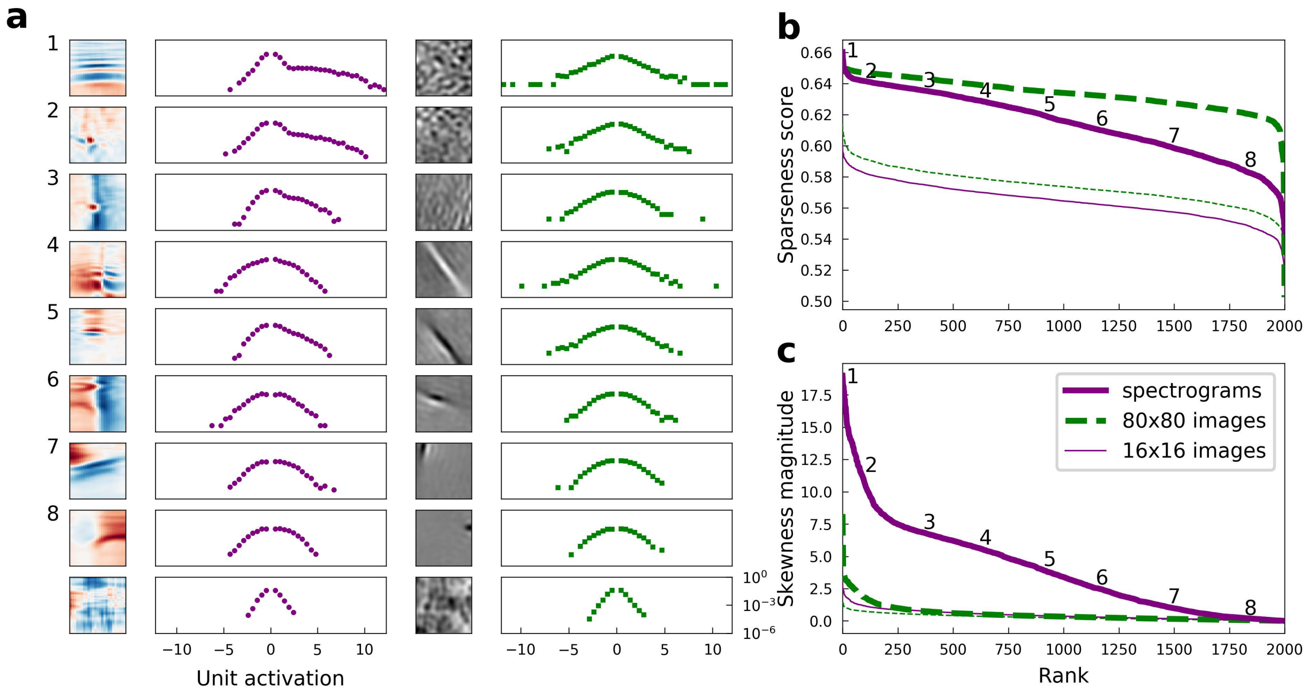

Figure S2. Overcomplete models exhibit some of the same contrasts observed in complete models. Same statistics as in Fig. 2 evaluated for 10-times overcomplete models. Although there is no clear clustering of auditory feature types as observed in the complete regime, the auditory features continue to show a greater diversity of sparseness values and activity distribution shapes. Note that the full auditory dictionary is shown in Fig. S12 and that the examples in panel **a** do not represent all the feature types in the overcomplete spectrogram dictionary.

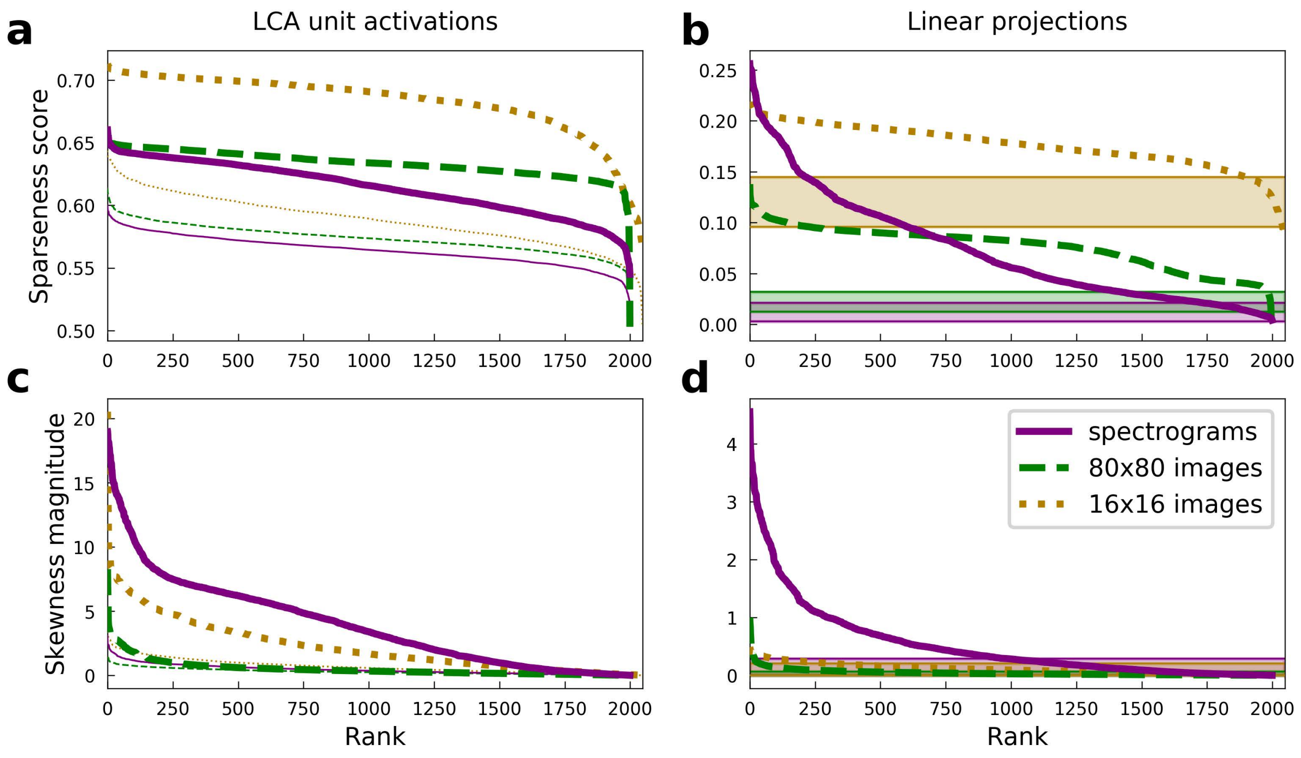

Figure S3. Statistics of highly overcomplete sparse representations for spectrograms and images with both preprocessing schemes. This figure is identical to Fig. 3 except for the addition of a third dataset consisting of filter-whitened 16x16 image patches (beige).

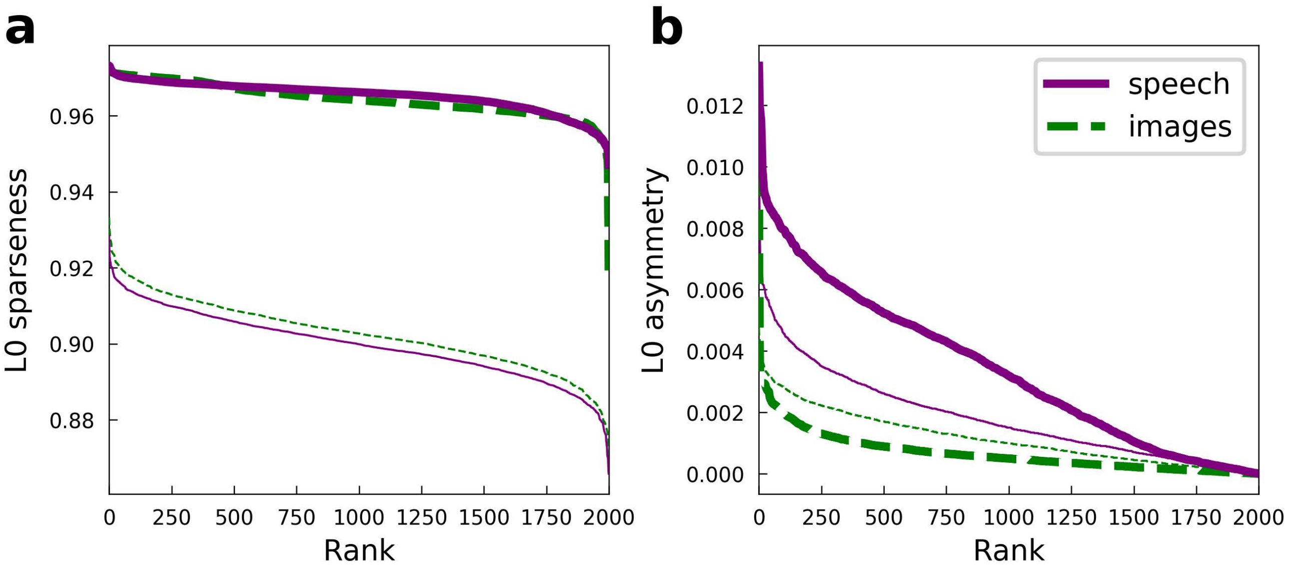

Figure S4. Statistics of highly overcomplete sparse representations for spectrograms and images: using L0 norm. This figure is identical to Fig. 3a,c except that here L0 sparseness rather than L1 sparseness is plotted in panel a, and the asymmetry in panel b is quantified as the absolute value of the difference between the fraction of inputs that gave a positive response for a given unit and the fraction that gave a negative response. Note that, unlike what we found for L1 sparseness, the range of values for L0 sparseness are nearly identical for the image and speech models. However, the "L0 asymmetry" we plot in panel b is still much greater for typical speech units compared with image units, just as we found using other measures of asymmetry.

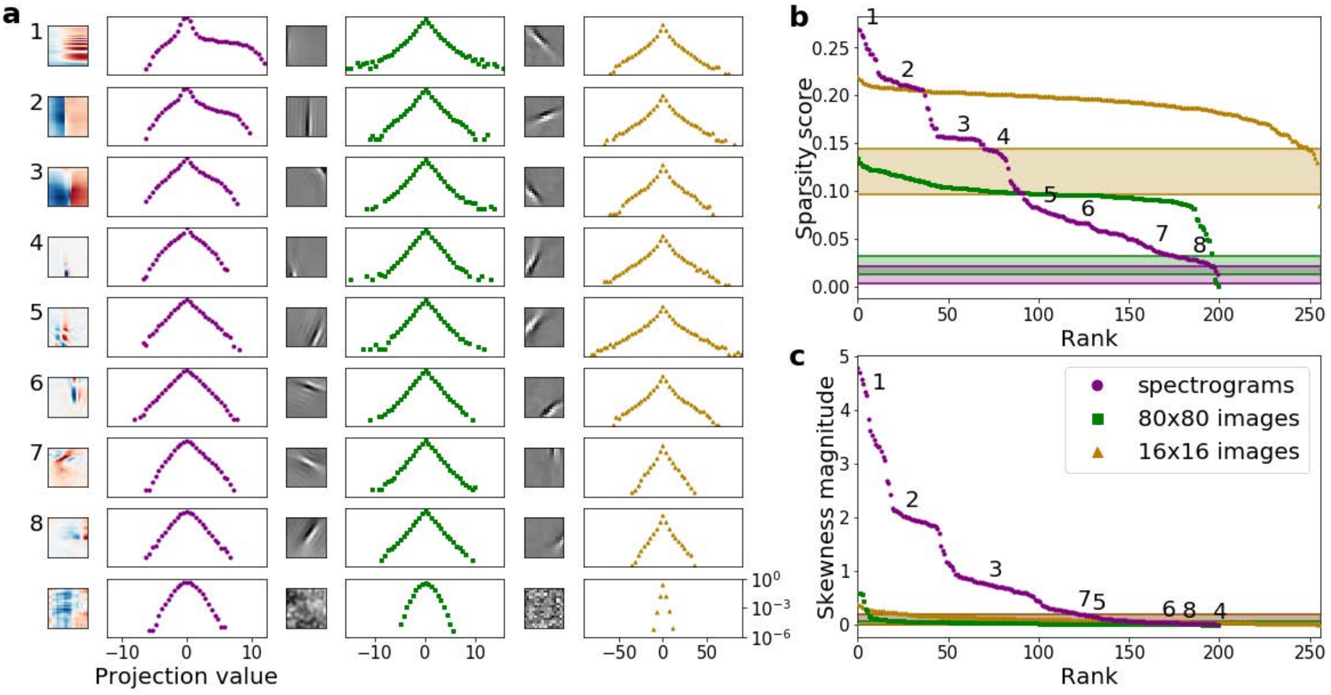

Figure S5. ICA learns a similar representation to complete sparse coding. This figure is identical to Fig. 7 except that the dictionaries have been learned with ICA.

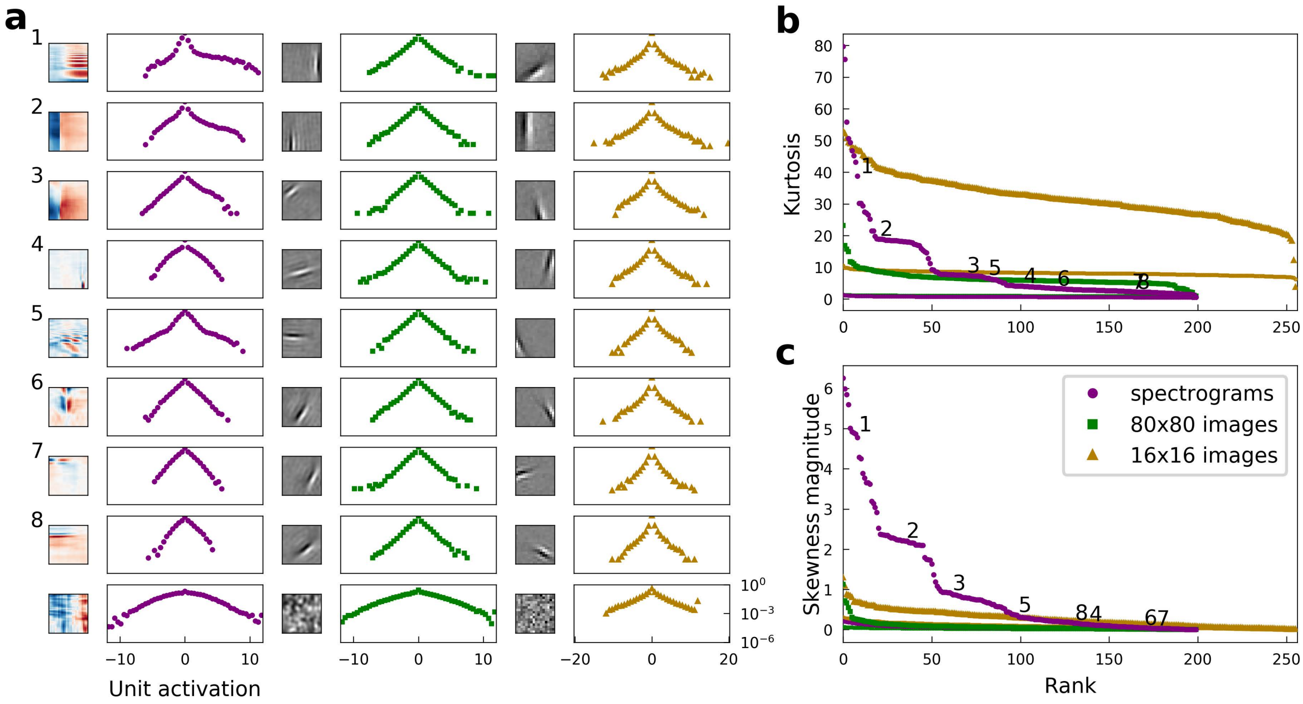

Figure S6. Using kurtosis to measure sparseness gives qualitatively similar results This figure is identical to Fig. 2 except that in panel **b** the sparseness score is replaced by the normalized fourth moment, *i.e.*, kurtosis.

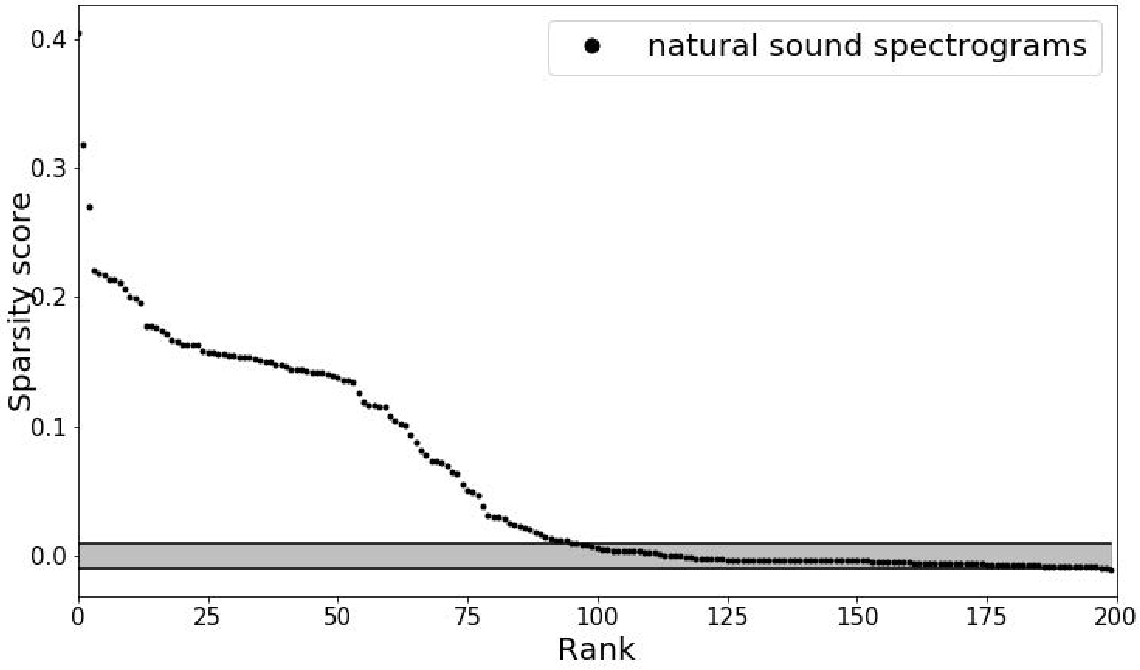

Figure S7. Sparseness score rank plot for natural sounds dictionary Sparseness scores for the dictionary elements in Fig. S20. Many of the sparseness scores are within the same range as random directions.

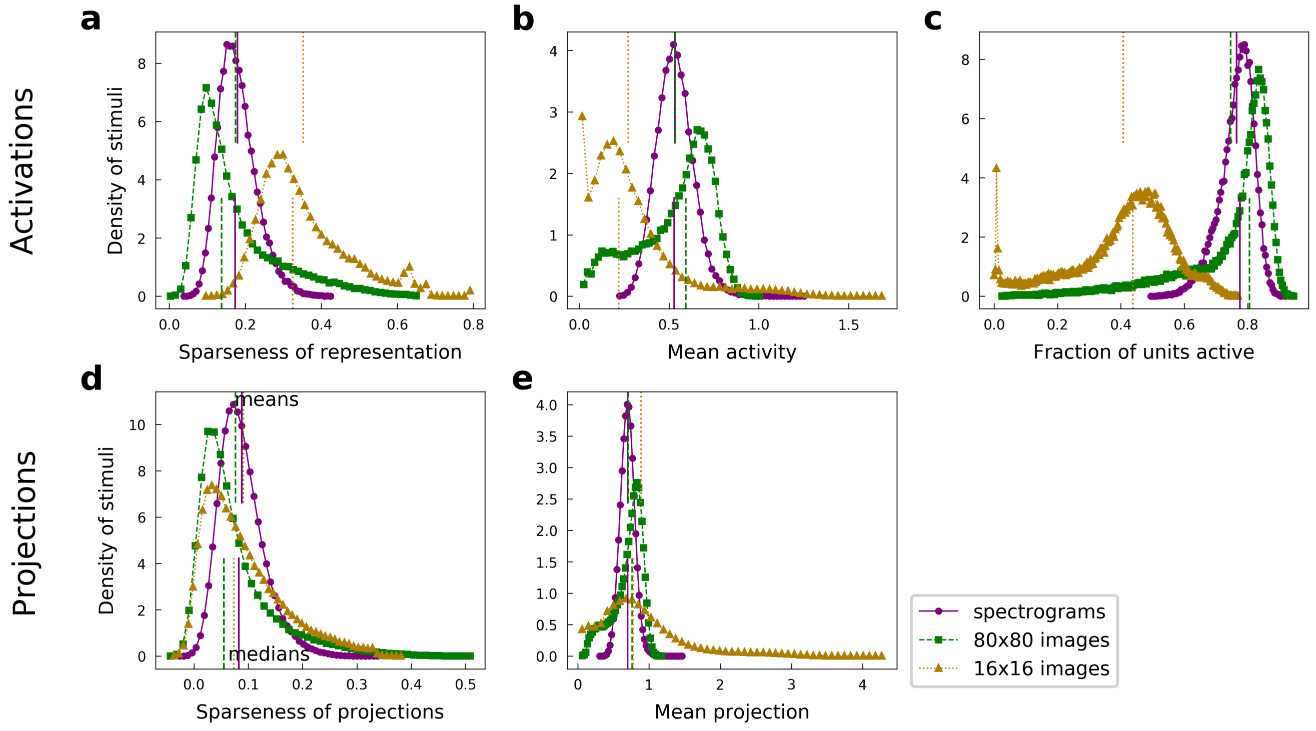

Figure S8. Population sparseness for complete models This figure is identical to Fig. 8 except the activations are computed with our complete sparse coding models.

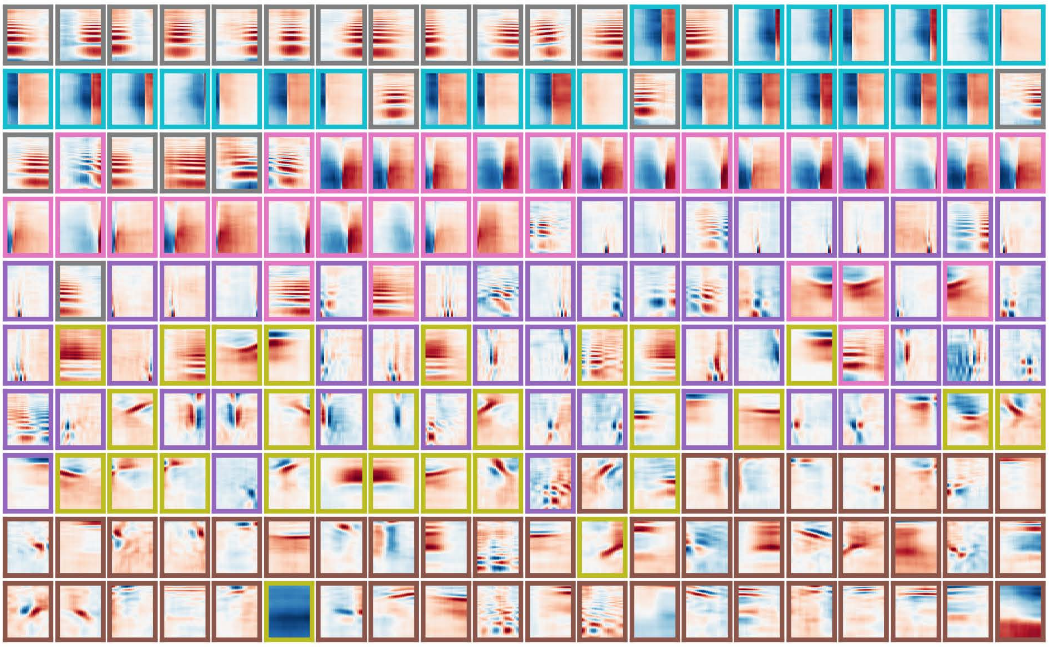

Figure S9. Complete sparse coding dictionary for the speech spectrogram dataset. There are 200 elements, spanning the 200-dimensional space of retained principle components. The elements are ordered from most sparse (upper left) to least sparse (lower right) unit activations. Color and scaling conventions are the same as in the main figures; each element is scaled independently before plotting. The border colors correspond to cluster labels. We fit a Gaussian mixture model with six components to the units' sparseness scores and skewness magnitudes. The labels are the most likely mixture component for each unit. Note that the dictionary elements are not used directly in the clustering; rather the clusters are determined by activation statistics. The first three of these clusters (gray, teal, and pink outlines) correspond roughly to the described qualitative clusters in the main text.

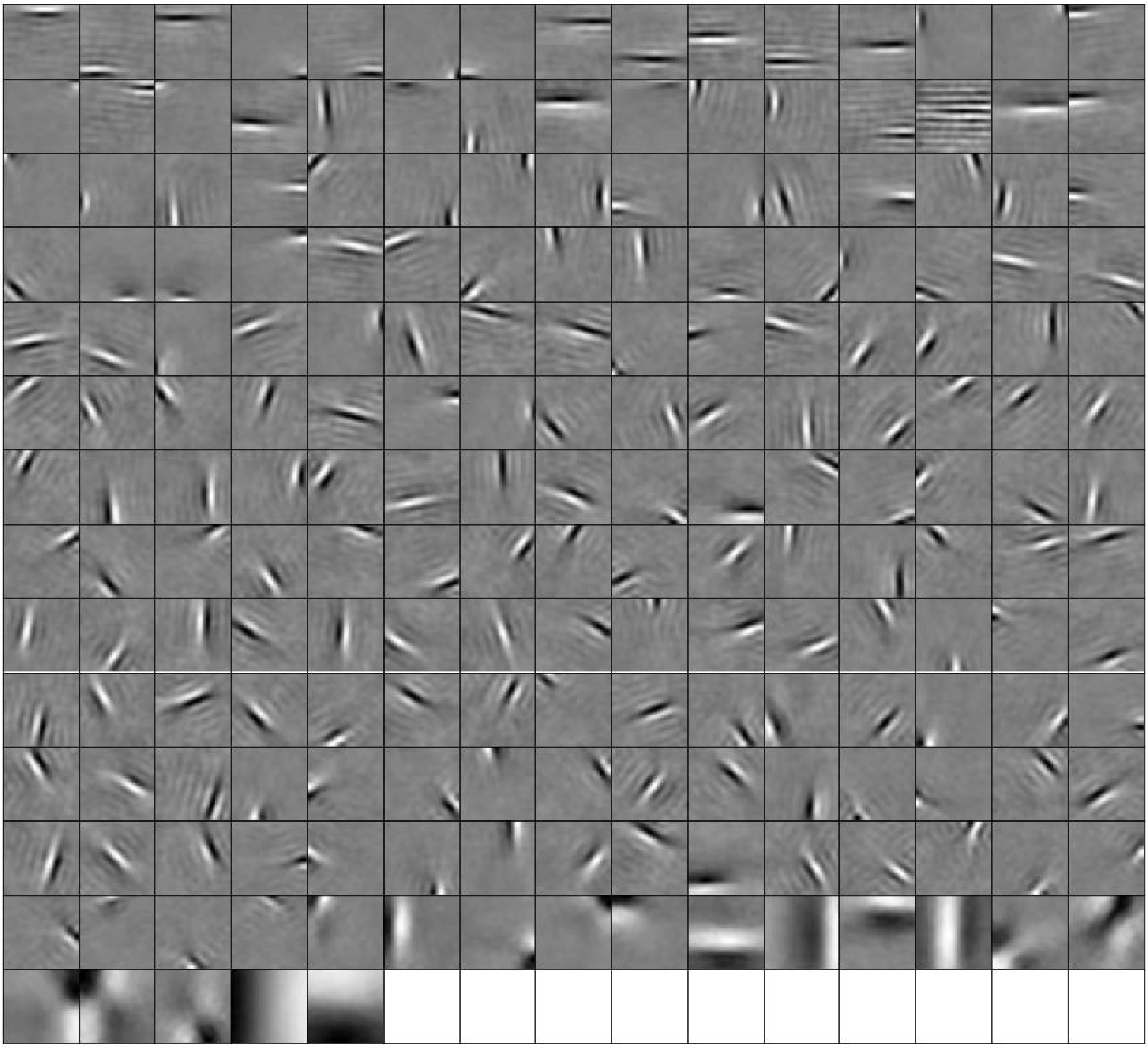

Figure S10. Complete sparse coding dictionary for the 80x80 pixel PCA-whitened image dataset. Sorted as in Fig. S9.

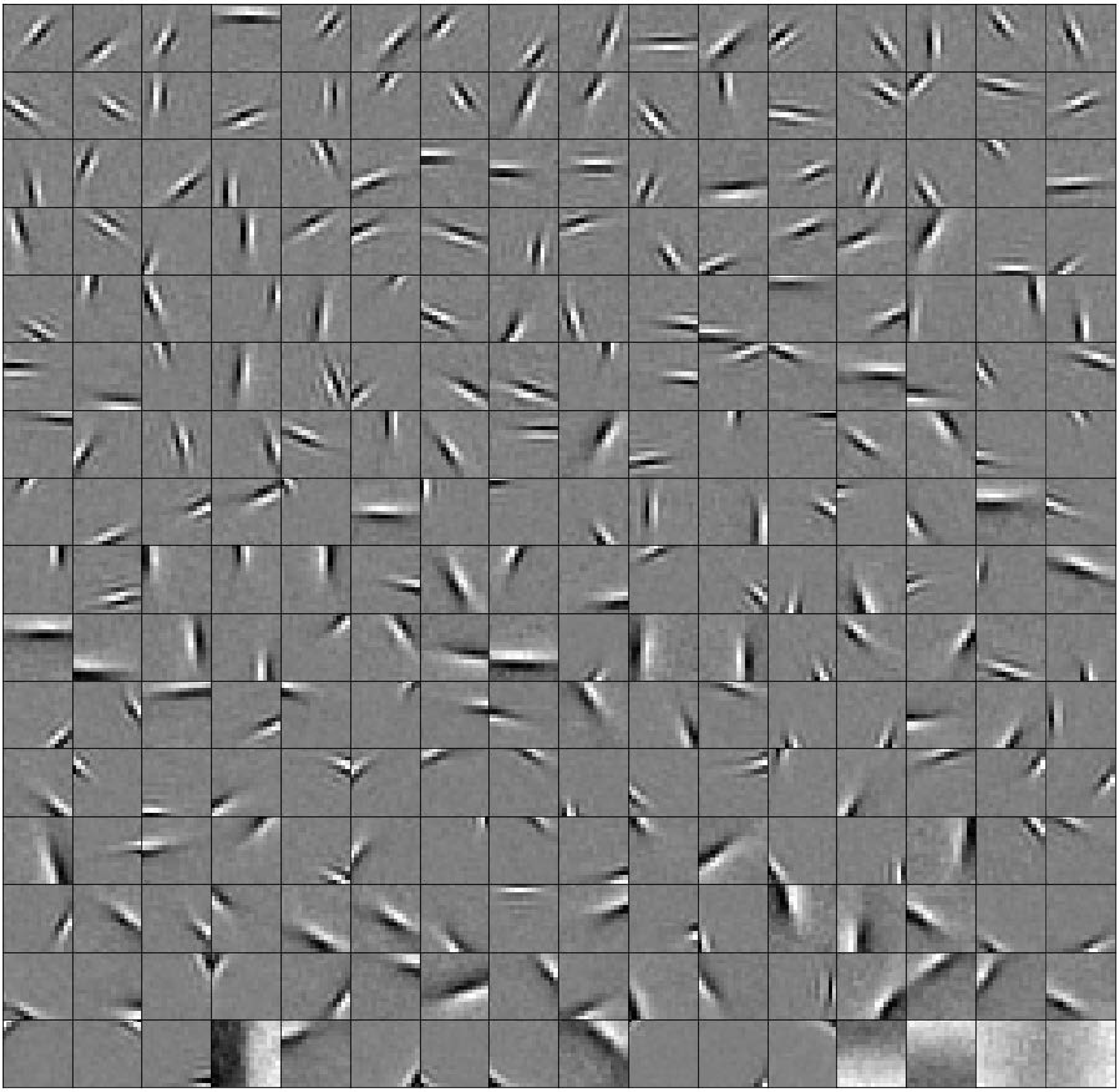

Figure S11. Complete sparse coding dictionary for the 16x16 pixel filter-whitened image dataset. Sorted as in Fig. S9.

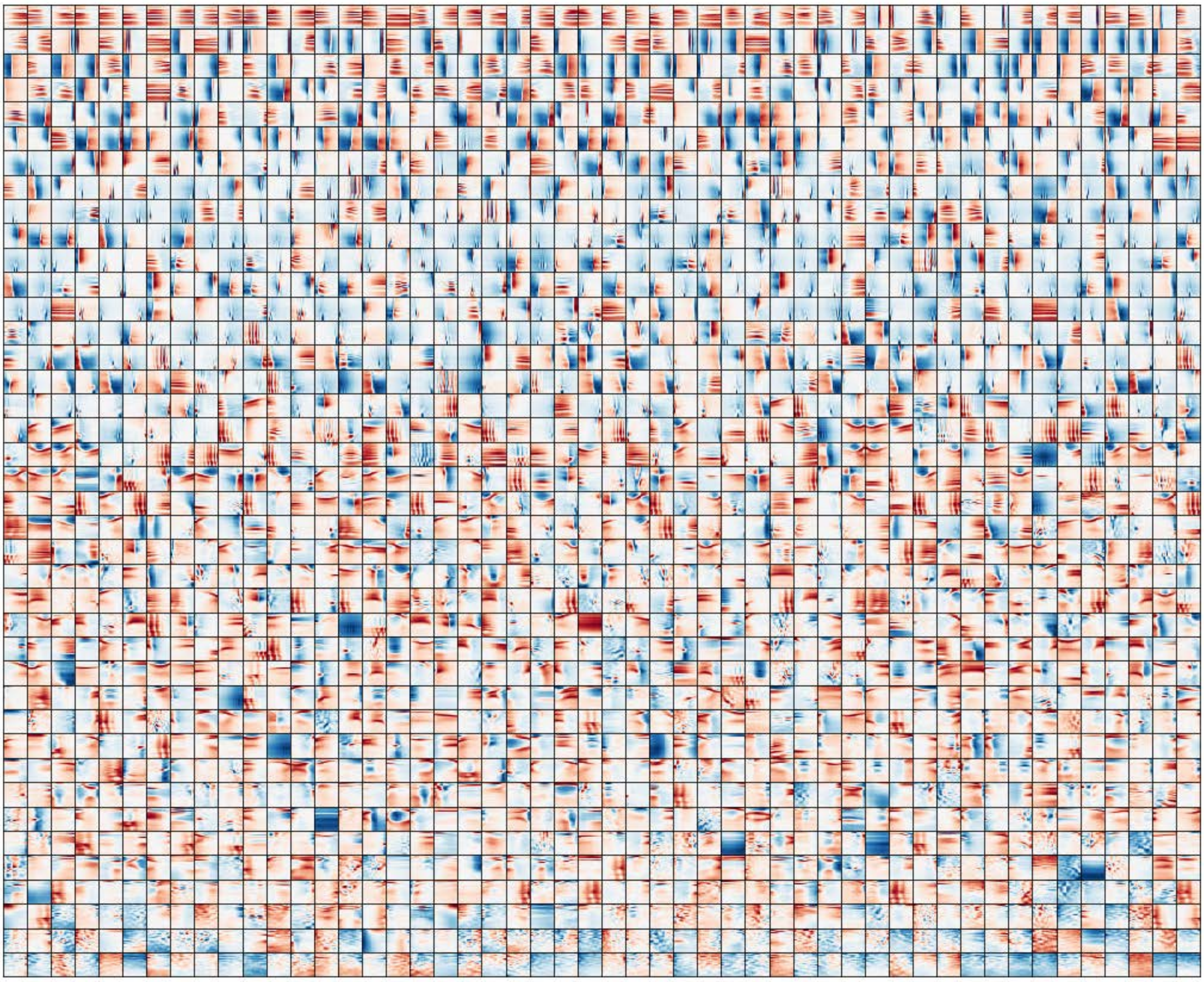

Figure S12. 10-times overcomplete sparse coding dictionary for the speech spectrogram dataset. There are 2000 dictionary elements, 10 times the number of retained principle components. These elements are not all mutually orthogonal. Sorted by sparseness of dot products with the data, as in Fig. S9. Note that this is not the same as sorting by LCA activities.

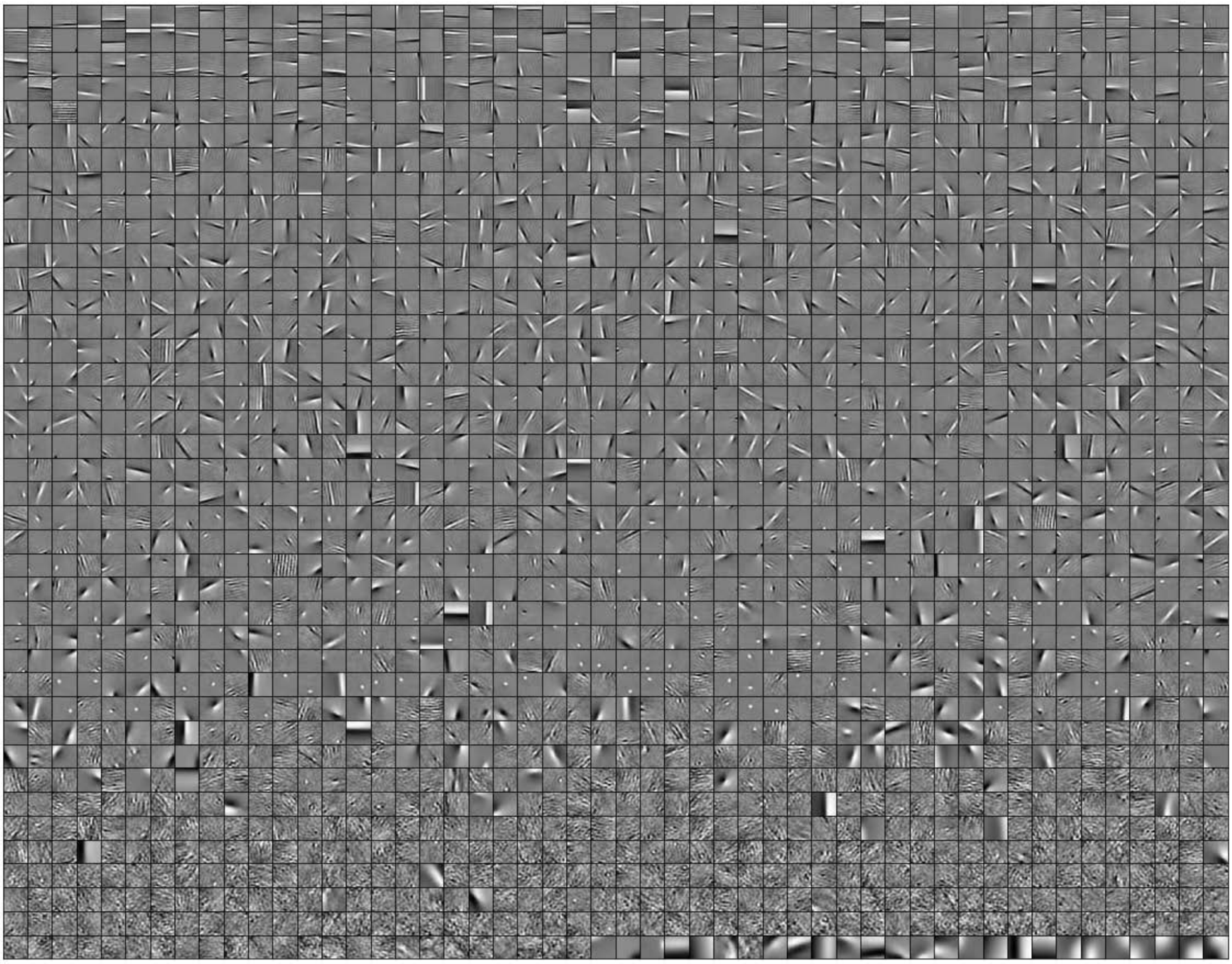

Figure S13. 10-times overcomplete sparse coding dictionary for the 80x80 pixel PCA-whitened image dataset. There are 2000 dictionary elements, 10 times the number of retained principle components. These elements are not all mutually orthogonal. Sorted as in Fig. S12.

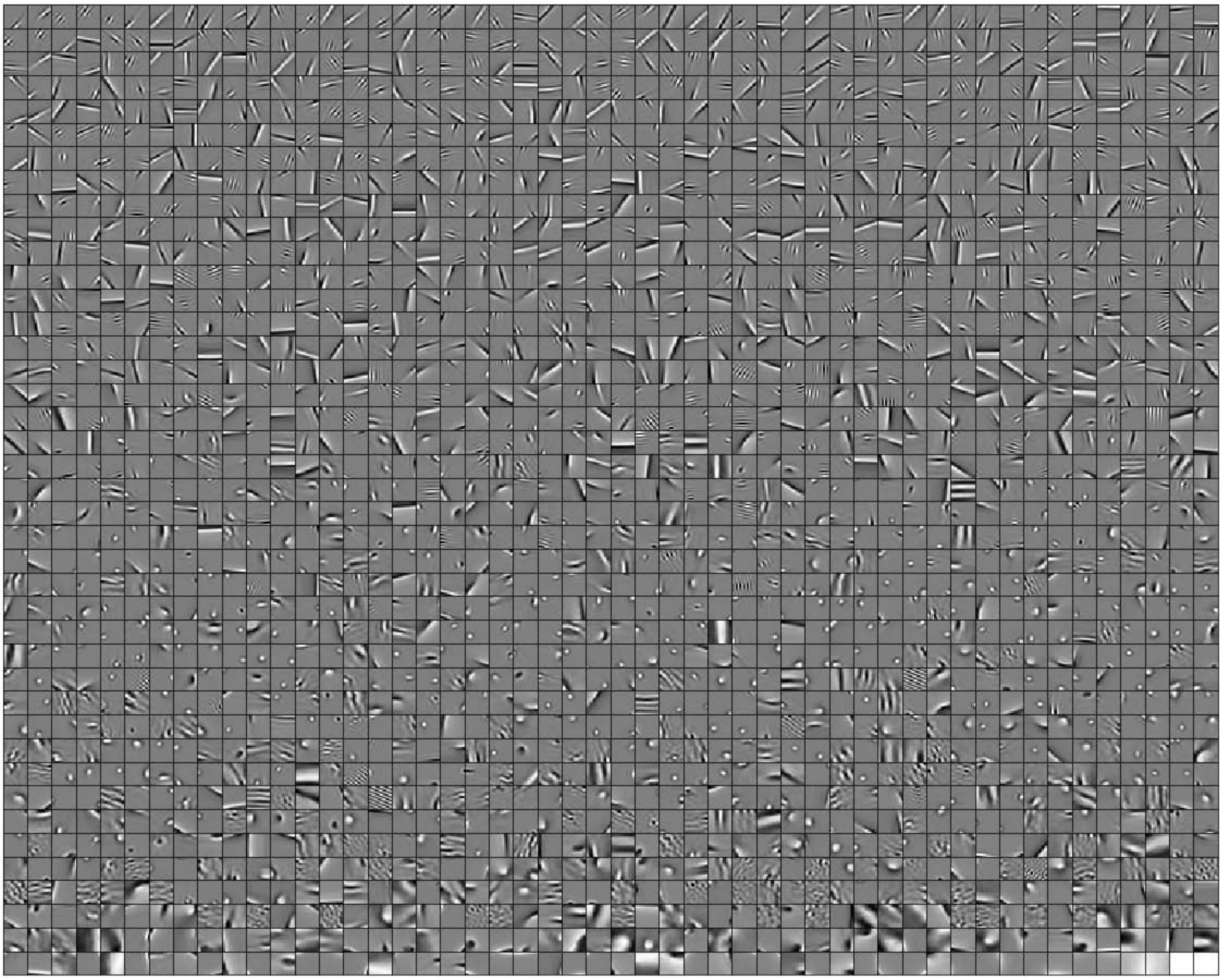

Figure S14. Approximately ten-times overcomplete sparse coding dictionary for the 16x16 pixel filter-whitened image dataset. There are 2048 dictionary elements, 8 times the number of pixels (256). While the dictionary is therefore nominally 8-times overcomplete, lowpass filtering cut the number of significant dimensions to about 200, making this dictionary approximately 10-times overcomplete. The elements are not all mutually orthogonal. Sorted as in Fig. S12.

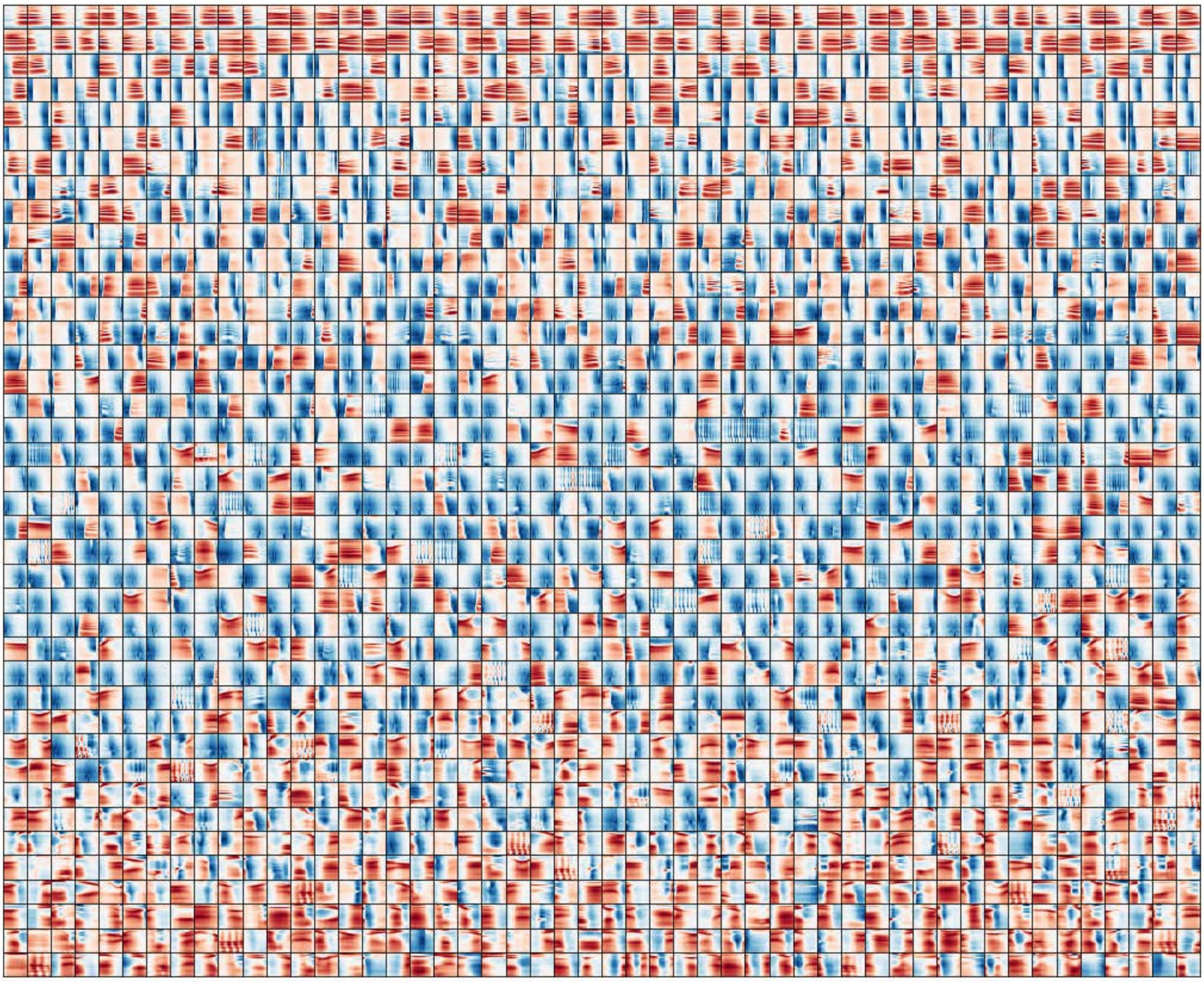

Figure S15. Ten-times overcomplete SAILnet dictionary for the speech spectrogram dataset. SAILnet has additional learned parameters, which act to enforce sparseness and decorrelation. These parameters are not shown but have an effect on the learning process for the dictionary. Sorted as in Fig. S12.

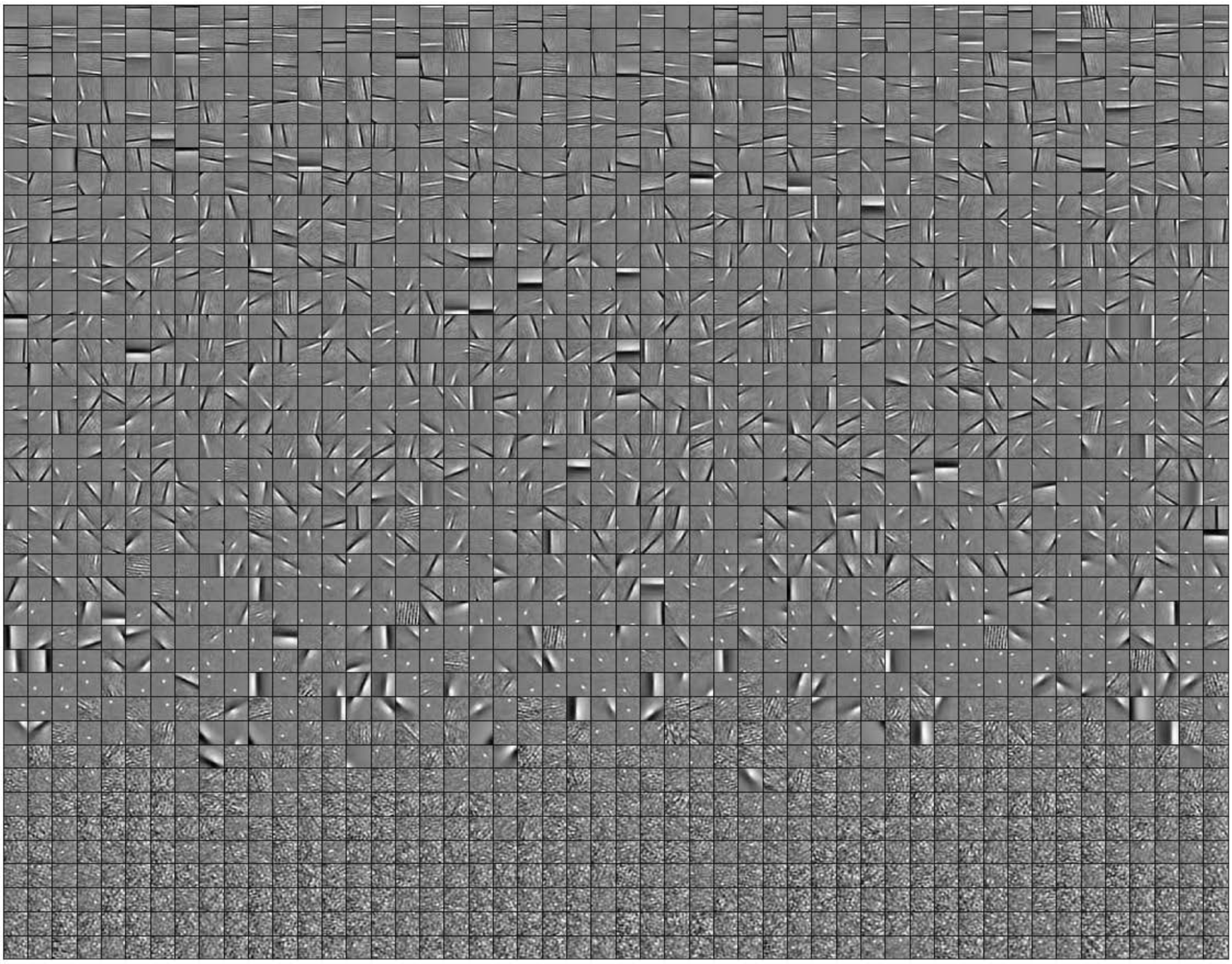

Figure S16. Ten-times overcomplete SAILnet dictionary for the 80x80 pixel PCA-whitened image dataset. Sorted as in Fig. S12.

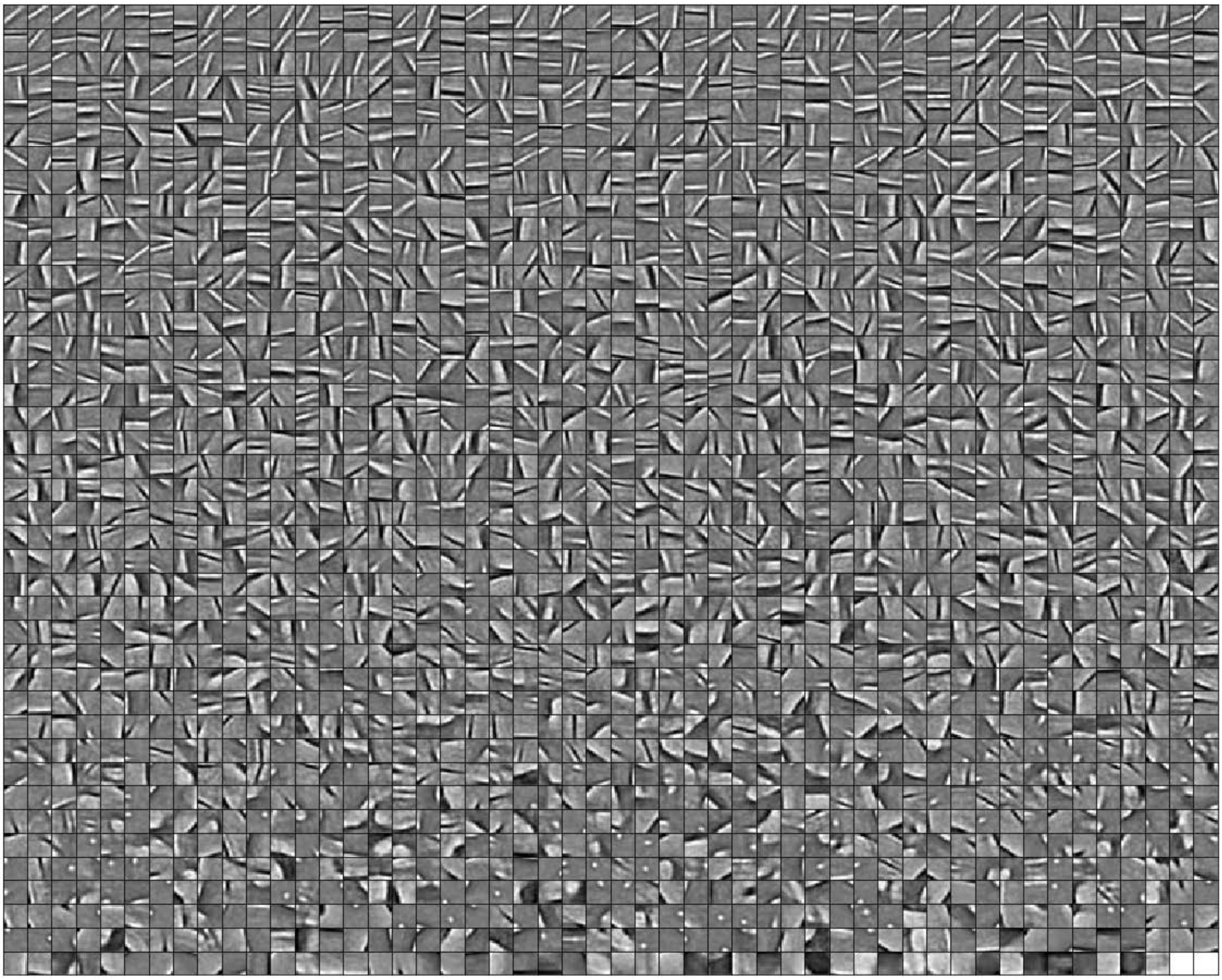

Figure S17. Approximately ten-times overcomplete SAILnet dictionary for the 16x16 pixel filter-whitened image dataset. Sorted as in Fig. S12.

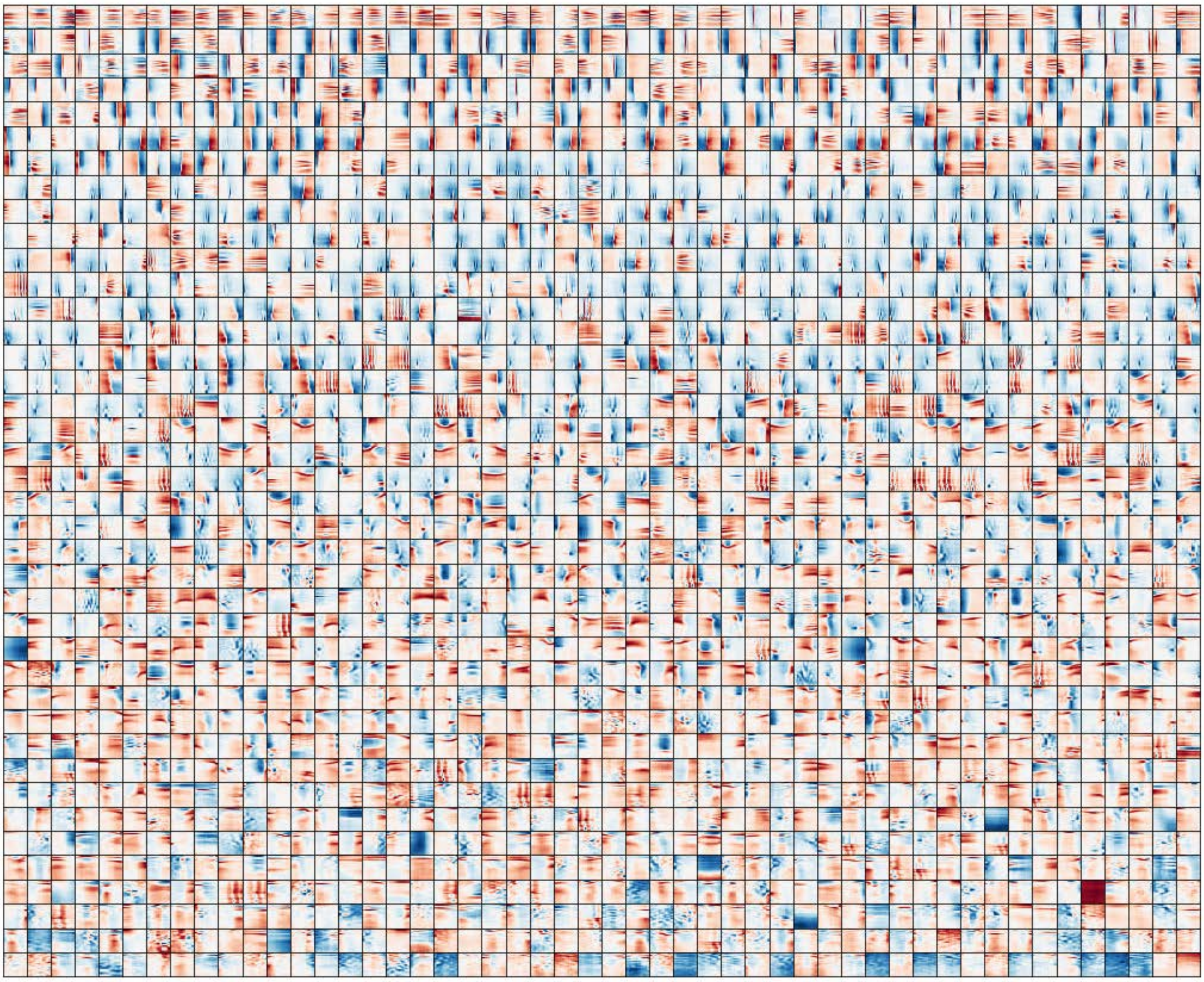

Figure S18. Ten-times overcomplete sparse coding dictionary for the speech spectrogram dataset, learned with non-negative activities. This dictionary was learned by the same procedure as the one in Fig. S12, but with the LCA thresholding function rectified so that negative activities instead set to zero. Sorted as in Fig. S12.

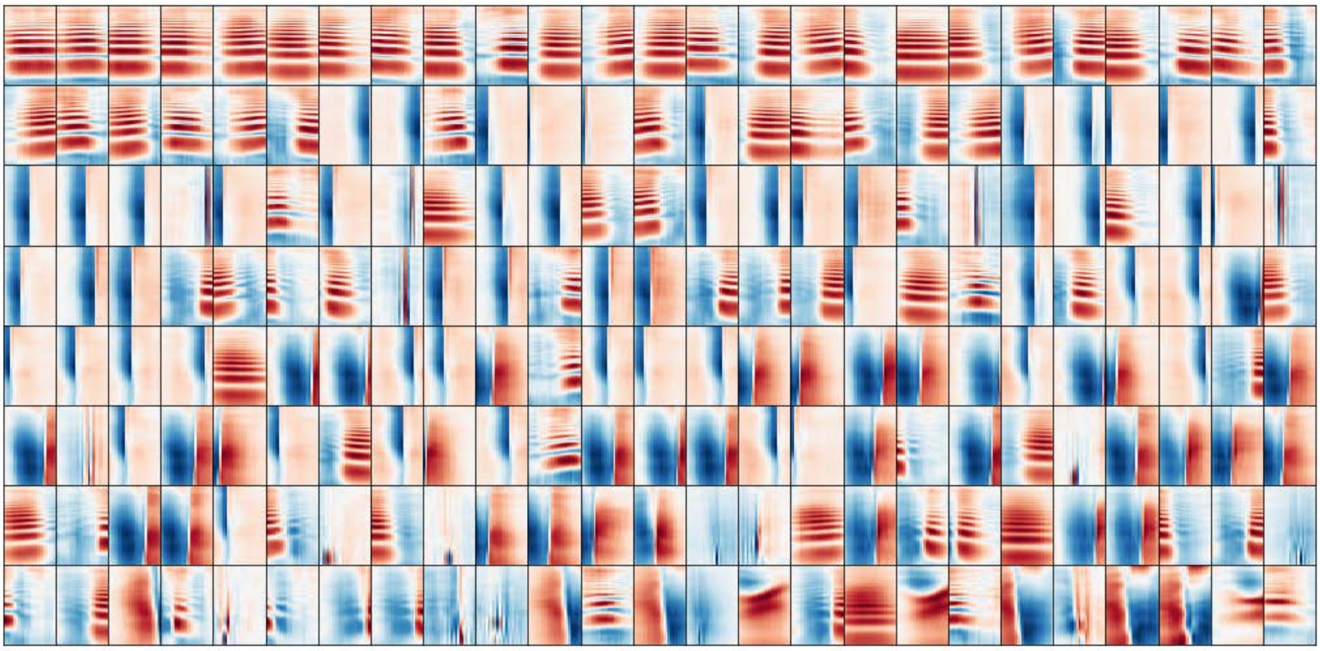

Figure S19. Complete dictionary for the speech spectrogram dataset, learned with modified SAILnet. This dictionary was learned with a version of SAILnet modified to allow negative spikes (which still count positively toward the average firing rate p). The dictionary has a different distribution of types with this modification, including the appearance of several elements with harmonic structure that changes sign abruptly in time. This type is not present in conventional SAILnet dictionaries, even at high degrees of overcompleteness. Sorted as in Fig. S12.

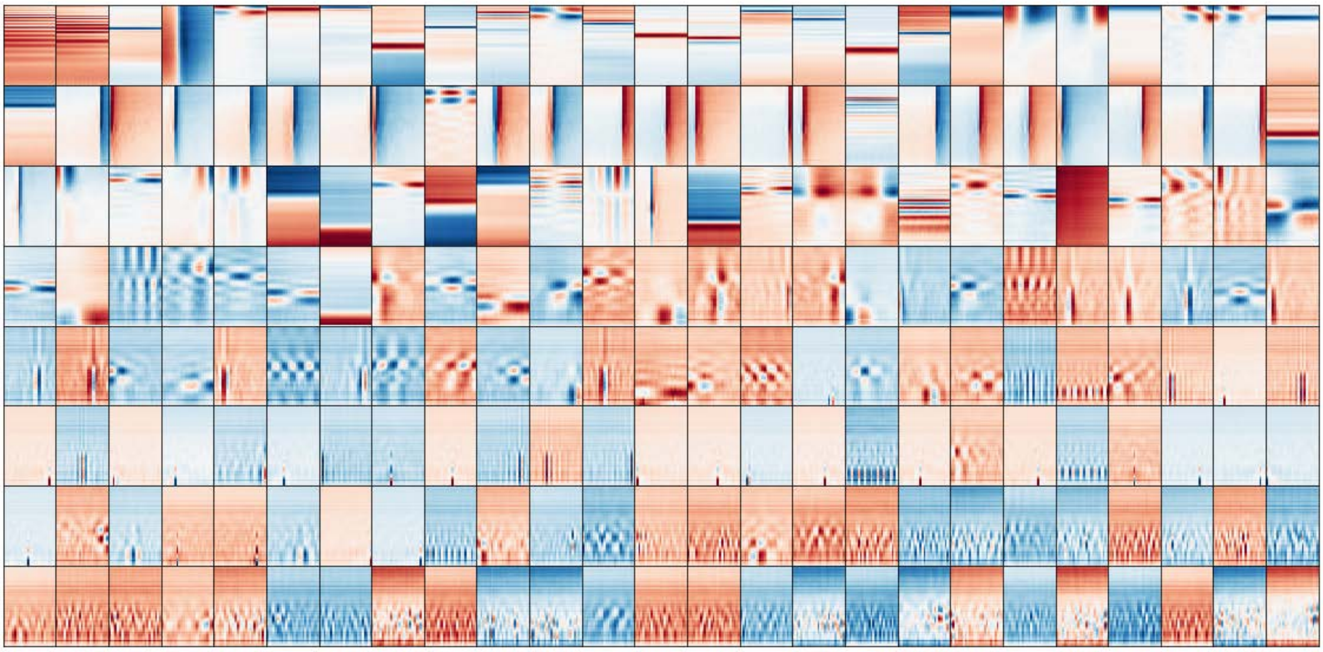

Figure S20. ICA dictionary fit to "natural sounds" dataset This dictionary was fit to a combination of natural sounds including ambient sounds recorded in various locations, animal vocalizations. It includes a few sparse elements reminiscent of harmonic stacks, single frequency detectors, and checkerboard-like features also seen in the speech-trained dictionaries. The majority of the elements, however, are no more sparse than random directions and have no easily discernible structure. Overcomplete sparse coding dictionaries trained on the same data show similar structure. Sorted as in Fig. S9.

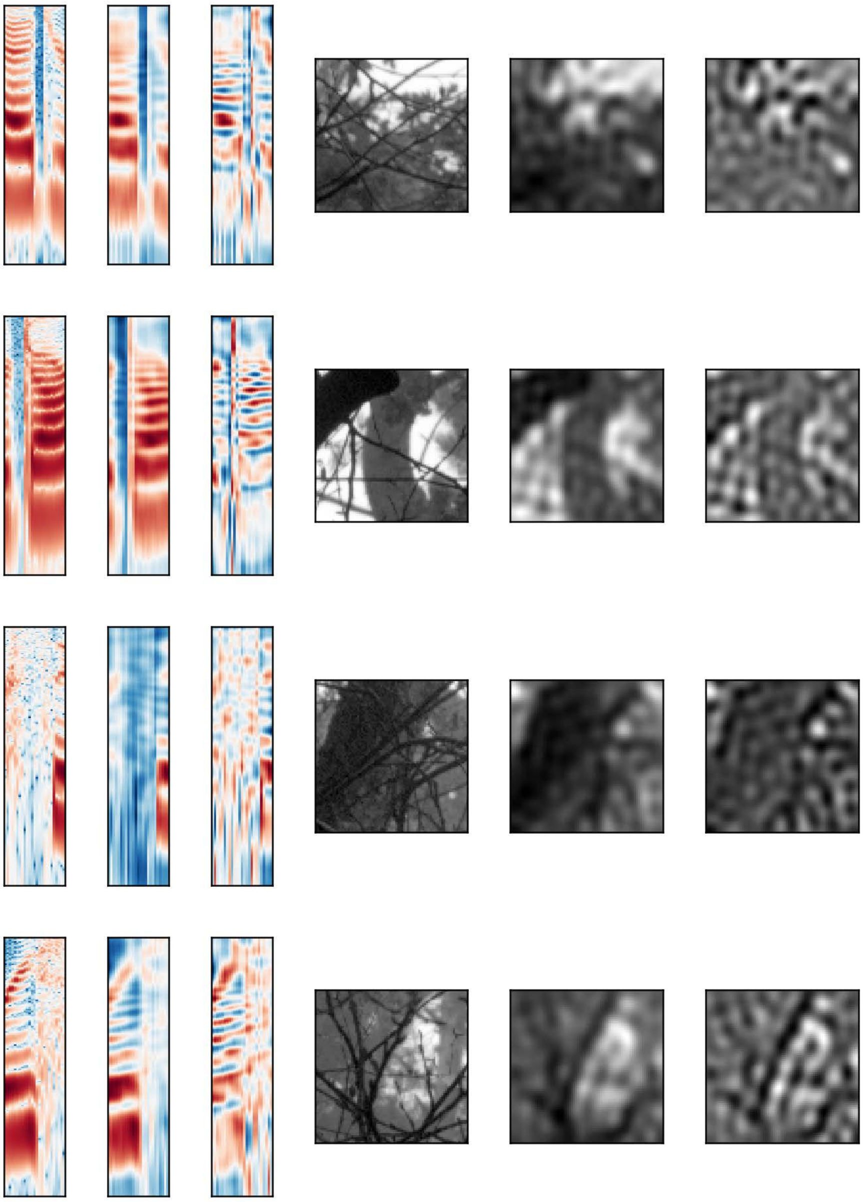

Figure S21. Data reconstruction from 200 principal components. Each triple shows an original spectrogram or image patch followed by its reconstruction from 200 principal components and then by its reconstruction from 200 principal components after whitening. While reconstructions from the reduced data are clearly distinguishable from the original data, they retain much of the original structure, particularly at coarse scales. The speech in the audio reconstructions is distorted but comprehensible.
